# Crystal structures of the CPAP/STIL complex reveal its role in centriole assembly and human microcephaly

**DOI:** 10.7554/eLife.01071

**Published:** 2013-09-17

**Authors:** Matthew A Cottee, Nadine Muschalik, Yao Liang Wong, Christopher M Johnson, Steven Johnson, Antonina Andreeva, Karen Oegema, Susan M Lea, Jordan W Raff, Mark van Breugel

**Affiliations:** 1 Sir William Dunn School of Pathology, University of Oxford, Oxford, United Kingdom; 2 Department of Cellular and Molecular Medicine, Ludwig Institute for Cancer Research, University of California, San Diego, La Jolla, United States; 3 Laboratory of Molecular Biology, Medical Research Council, Cambridge, United Kingdom; Howard Hughes Medical Institute, University of California, Berkeley, United States

**Keywords:** centriole, centrosome, CPAP, microcephaly, STIL, *C. elegans*, *D. melanogaster*, Zebrafish

## Abstract

Centrioles organise centrosomes and template cilia and flagella. Several centriole and centrosome proteins have been linked to microcephaly (MCPH), a neuro-developmental disease associated with small brain size. CPAP (MCPH6) and STIL (MCPH7) are required for centriole assembly, but it is unclear how mutations in them lead to microcephaly. We show that the TCP domain of CPAP constitutes a novel proline recognition domain that forms a 1:1 complex with a short, highly conserved target motif in STIL. Crystal structures of this complex reveal an unusual, all-β structure adopted by the TCP domain and explain how a microcephaly mutation in CPAP compromises complex formation. Through point mutations, we demonstrate that complex formation is essential for centriole duplication in vivo. Our studies provide the first structural insight into how the malfunction of centriole proteins results in human disease and also reveal that the CPAP–STIL interaction constitutes a conserved key step in centriole biogenesis.

**DOI:** http://dx.doi.org/10.7554/eLife.01071.001

## eLife digest

Organisms—and individual tissues—grow and develop by dividing their cells. However, the process of cell division does not have to be symmetric, and the fates of the cells can be very different if cellular contents, including RNAs or proteins, are exclusively retained in the ‘mother’ or passed to her ‘daughter’. Organelles known as centrioles can play an important part in influencing whether cell division is symmetric or asymmetric.

Centrioles contain ordered assemblies of various proteins, and mutations in some of these proteins can cause developmental defects in humans. For example, mutations in the centriolar proteins CPAP and STIL cause a syndrome known as microcephaly, in which the brain is smaller than normal. Although CPAP and STIL are known to bind each other, how they interact on a molecular level to form centrioles—and how this interaction is disrupted in microcephaly—is not well understood.

Cottee et al. have now used structural and biochemical assays to explore how these two proteins bind to each other, and have identified specific amino acid residues that enable this interaction. These residues are highly conserved across many organisms, and a mutation in one of them has previously been associated with microcephaly in humans. Now, Cottee et al. demonstrate that this mutation weakens the interaction between CPAP and STIL in vitro.

To explore these processes in vivo, Cottee et al. studied mutant fruit flies in which the interactions between CPAP and STIL were weaker than normal, and found that these mutations prevented the normal formation of centrioles. Furthermore, there was a striking correlation between the ability to form centrioles in fruit flies and the ability of CPAP and STIL to bind each other, based on the structural model and in vitro binding studies.

Cumulatively, these findings reinforce the importance of CPAP and STIL in centriole formation, and suggest that one reason for the development of microcephaly may be defects in the proper formation of centrioles.

**DOI:** http://dx.doi.org/10.7554/eLife.01071.002

## Introduction

Centrioles are small cylindrical organelles whose outer walls contain a ninefold symmetric array of microtubule triplets. These structures form the basal bodies that template the assembly of cilia and flagella, and they also organise a proteinaceous matrix termed the pericentriolar material (PCM) to form centrosomes, the main microtubule organising centres in animal cells. These organelles play an important part in many aspects of cell organisation, and centriolar dysfunction is linked to a plethora of human diseases, including cancer, obesity, macular degeneration and polycystic kidney disease (Nigg and Raff, 2009; Bettencourt-Dias et al., 2011).

Recently, an unexpected genetic link has emerged between centriole/centrosome assembly and human brain size. Autosomal recessive primary microcephaly (MCPH) is a rare condition where patients are born with small brains (Thornton and Woods, 2009). All eight identified MCPH genes encode proteins that localise to centrioles and/or centrosomes/spindle poles (Thornton and Woods, 2009; Hussain et al., 2012). It is unclear why mutations in these proteins are linked to such a specific neuro-developmental problem in humans, but it seems likely that some aspect of centriole/centrosome function must be particularly important for the proper proliferation of human neural progenitors (Siller and Doe, 2009; Megraw et al., 2011). In support of this possibility, mutations in the centriolar components CPAP (DSas-4 in *Drosophila*, here called dCPAP) and STIL (Ana2 in *Drosophila*, here called dSTIL) in flies lead to defects in the asymmetric division of larval neural stem/progenitor cells (Basto et al., 2006). Mutations in MCPH proteins in mice, however, lead to complex phenotypes that can include, but are not restricted to, microcephaly (McIntyre et al., 2012). Moreover, compelling genetic links are now emerging between centrioles/centrosomes and DNA damage repair (DDR) pathways: mutations in certain MCPH genes and in genes encoding other centriole/centrosome proteins can lead to Seckel syndrome and MOPD, pathologies normally associated with defects in DDR (Megraw et al., 2011). Thus, the cellular mechanisms that lead to pathology when centriole/centrosome proteins are mutated in humans remain unclear.

Centrioles are complex structures, but work in several model systems revealed only a small number of conserved proteins to be important for centriole assembly. These include PLK4/SAK, SAS-6, STIL/Ana2, CPAP/CenpJ/SAS-4, Cep152/Asl, and CEP135 (Brito et al., 2012; Gonczy, 2012). Several studies have identified a complex web of putative interactions between these proteins (Cizmecioglu et al., 2010; Dzhindzhev et al., 2010; Hatch et al., 2010; Tang et al., 2011; Vulprecht et al., 2012; Lin et al., 2013). However, an understanding of centriole architecture and its assembly mechanisms will ultimately require high-resolution structures of the key centriolar components and their complexes. The power of combining structural studies with protein biochemistry and functional in vivo experiments has been demonstrated by work on SAS-6. These studies revealed how SAS-6 homo-oligomerises to organise the central cartwheel (Kitagawa et al., 2011b; van Breugel et al., 2011), the earliest structurally defined intermediate in centriole assembly (Brito et al., 2012; Gonczy, 2012), and suggested how SAS-6 might interact with SAS-5, the proposed STIL homologue in worms (Qiao et al., 2012). Additionally, high-resolution structures of Sak/Plk4 fragments have recently been solved (Leung et al., 2002; Slevin et al., 2012). However, equivalent studies with other core centriolar components or especially their complexes are currently missing, and how any of these proteins might be structurally and mechanistically compromised in MCPH is not known.

Of particular interest in this regard is the putative centriolar CPAP–STIL complex, as mutations in both components result in MCPH (Leal et al., 2003; Bond et al., 2005; Gul et al., 2006; Darvish et al., 2010). CPAP and STIL are strictly required for centriole assembly: STIL at a very early stage (Stevens et al., 2010b; Tang et al., 2011; Kitagawa et al., 2011a; Arquint et al., 2012; Vulprecht et al., 2012) and CPAP slightly later (Kirkham et al., 2003; Leidel and Gonczy, 2003; Basto et al., 2006; Kleylein-Sohn et al., 2007; Dobbelaere et al., 2008; Vulprecht et al., 2012), possibly by controlling the organisation (Pelletier et al., 2006; Dammermann et al., 2008) and length of the centriolar microtubules (Blachon et al., 2009; Kohlmaier et al., 2009; Schmidt et al., 2009; Tang et al., 2009; Kim et al., 2012). A direct interaction between STIL and CPAP has been observed in yeast-two-hybrid and pull-down experiments (Tang et al., 2011; Vulprecht et al., 2012). Intriguingly, a MCPH mutation (E1235V) in the conserved C-terminal domain of CPAP (the so-called TCP-domain or G-Box) appeared to weaken this yeast-two-hybrid interaction (Tang et al., 2011). Tissue culture experiments suggested that this MCPH mutation might cause a partial loss-of-function of CPAP (Kitagawa et al., 2011a). However, the same study also found that the E1235V mutation results in an enhanced functionality of CPAP when overexpressed in vivo (Kitagawa et al., 2011a). To understand how CPAP and STIL interact and how the MCPH mutation affects CPAP functionality in vitro and in vivo, we undertook a detailed biochemical, structural and functional study of the putative CPAP–STIL complex.

## Results

### The CPAP TCP domain binds to a conserved proline-rich motif in STIL

**Figure 1.**
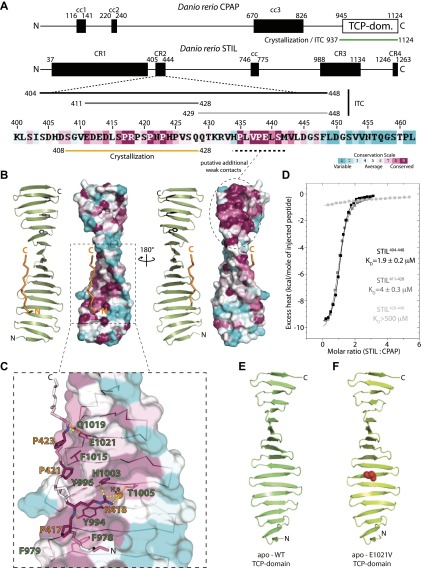
Biochemical and structural characterisation of the CPAP TCP domain and its interaction with STIL. (**A**) Schematic representation of *D. rerio* CPAP and STIL. CPAP is a 1124 amino acid (aa) protein with three predicted coiled coil (cc) domains and a C-terminal TCP domain. STIL is a 1263 aa protein with one predicted cc domain and several conserved regions (CR). The proline-rich CR2 domain is enlarged and coloured according to Consurf conservation scores (Glaser et al., 2003) from cyan (variable) to burgundy (conserved). The constructs used in this study are indicated by bars. (**B**) Two views of the TCP domain structure (green) in complex with the STIL peptide (orange), rotated by 180°. Images on the left of each view show a ribbon representation and images on the right show the TCP domain as a molecular surface coloured according to Consurf conservation scores. Note the presence of a conserved patch (dashed circle) along the edge of the TCP domain where the STIL peptide is bound. This patch contains aromatic residues (black sticks) that would be well placed to interact with conserved prolines in the C-terminal part of the STIL CR2 region that we had to omit for crystallisation. ITC experiments (Figure 1D) suggest that these putative additional contacts would only contribute weakly to overall binding. (**C**) Detailed view of the *D. rerio* CPAP–STIL interaction interface coloured according to Consurf conservation scores. Interface residues are shown in sticks, and the TCP domain is shown as a semi-transparent molecular surface. Contact residues are labelled in green (CPAP) and orange (STIL). Dotted yellow lines indicate hydrogen-bonds. The dark orange sphere represents a bound water molecule. (**D**) ITC analysis using the STIL constructs shown in Figure 1A. The excess heat measured on titrating STIL into CPAP at 25°C was fitted to a single set of binding sites model. Fitted K_D_ values are indicated together with their standard deviations. (**E** and **F**) Ribbon models of the apo-structures of the *D. rerio* CPAP TCP domain: (**E**) WT apo-structure; (**F**) E1021V (MCPH mutation) apo-structure (V1021 represented as red spheres). **DOI:** http://dx.doi.org/10.7554/eLife.01071.003

Yeast-two-hybrid experiments suggested that a region of human CPAP comprising its conserved C-terminal TCP domain (or G-box) can interact with a ∼400 amino acid (aa) region (residues 231–619) of human STIL (Tang et al., 2011). To try to identify the region of STIL most likely to be involved in an interaction with CPAP, we carried out a sequence alignment with multiple metazoan STIL proteins (Figure 1A, Figure 1—figure supplement 1). This analysis revealed a short (∼40 aa) highly conserved proline-rich region (CR2) (Figure 1A) within this interval. To test whether this region of STIL could bind to the CPAP TCP domain, we recombinantly produced the TCP domain of *Danio rerio* CPAP and used isothermal titration calorimetry (ITC) to test its ability to bind to a fragment of *D. rerio* STIL that spanned CR2 (residues 404–448) (Figure 1D, Table 1). The two proteins formed a 1:1 complex with a K_D_ of ∼2 μM. Next, we further split the peptide to test the binding contribution from its N-terminal (residues 411–428) and C-terminal region (residues 429–448). The N-terminal region exhibited an only slightly weaker binding (K_D_ ∼4 μM) to the TCP domain, whereas the C-terminal region showed a very weak binding (K_D_ > 500 μM) (Figure 1D; Table 1). We conclude that the CPAP TCP domain binds to a short conserved motif in STIL (CR2) with a potentially biologically significant affinity, and that the majority of the binding affinity comes from interactions with residues within the first proline-rich region in CR2.

**Figure 1—figure supplement 1.**
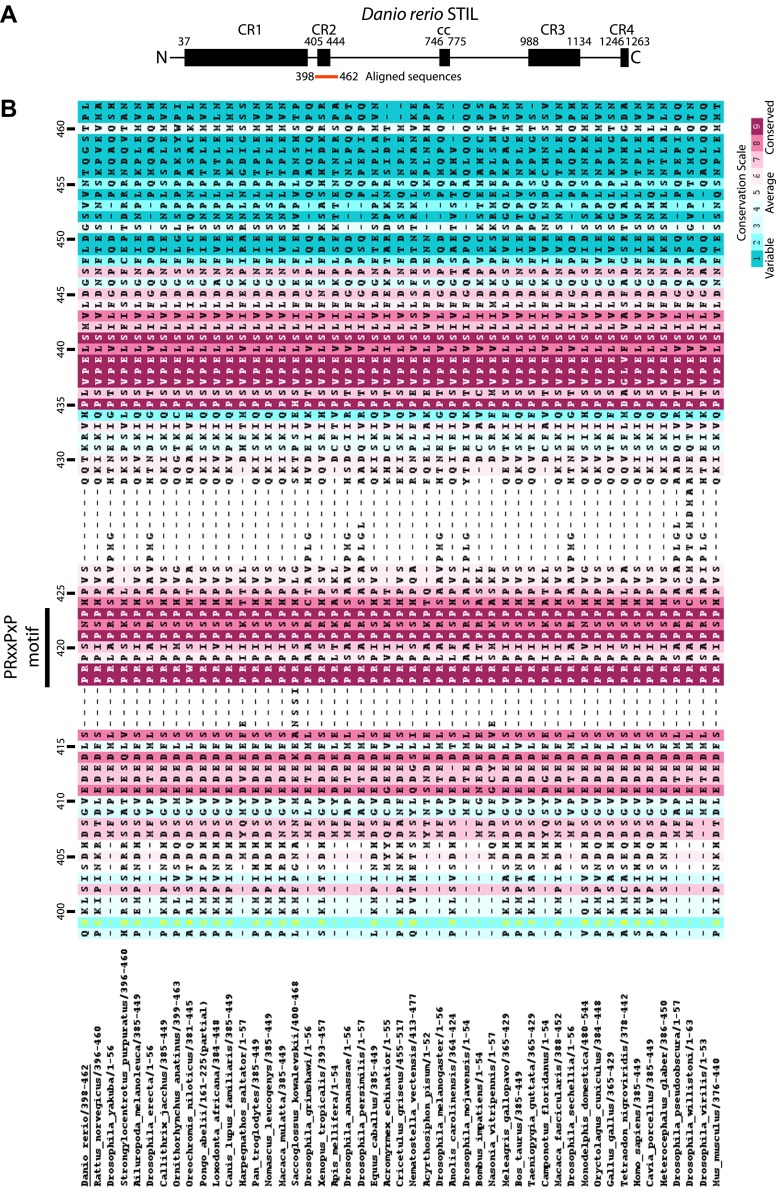
Multiple sequence alignment of the conserved proline-rich region of STIL (CR2). The numbering refers to *D. rerio* STIL. The alignment is coloured by conservation according to the Consurf conservation score from cyan (variable) to burgundy (conserved). **DOI:** http://dx.doi.org/10.7554/eLife.01071.004

**Figure 1—figure supplement 2.**
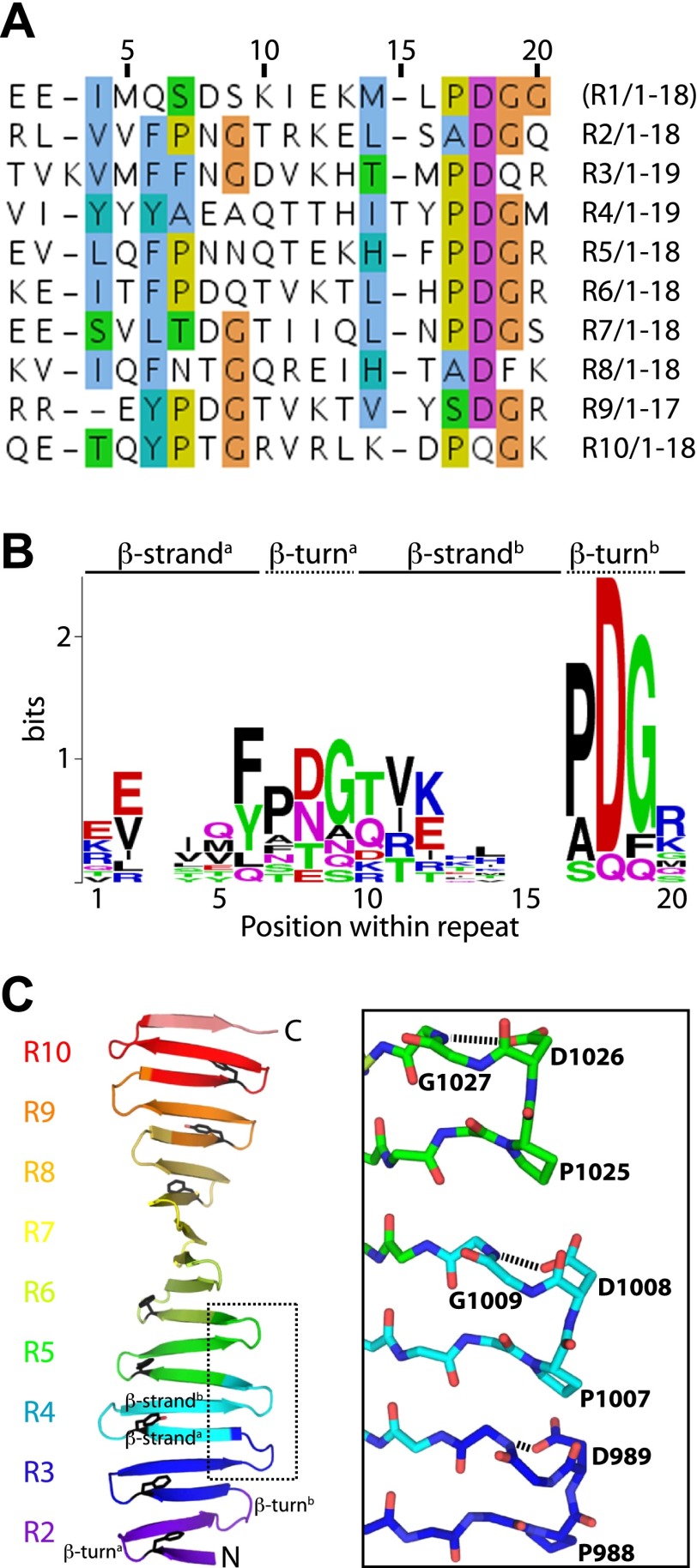
The TCP domain sequence repeats. (**A**) Alignment of the sequence repeats of *D. rerio* CPAP^937–1124^. Residues are coloured according to the Clustalx colour scheme. R, Repeat. Repeat 1 was not visible in the electron density map of *D. rerio* apo-CPAP^937–1124^ but could be seen partially in the structure of the complex between *D. rerio* CPAP^937–1124^ and STIL^408–428^. (**B**) Sequence logo of the CPAP^937–1124^ repeat with the relative residue frequencies at each position. Prominent features of this repeat are two PDG motifs and the high frequency of aromatic residues adjacent to the first PDG motif in position 6 of the repeat. (**C**) Left: ribbon presentation of the *D. rerio* apo CPAP^937–1124^ structure with its sequence repeats rainbow-coloured from N- to C-terminus. R, Repeat. An individual structural repeat consists of a β-hairpin. The aromatic residues found in position 6 of the repeat are shown in black sticks. These aromatic residues run along the edge of one side of the β-sheet, where the proline-rich STIL peptide binds. The PDG motifs frequently constitute the β-turns of the TCP repeats. Boxed are three of these turns that are presented on the right as a close-up. In this close-up, residues of the PDG motif are labelled and shown in sticks. The Asp residue in this motif hydrogen-bonds (dotted black lines) to the main-chain of the (n) + 1 neighbouring residue. **DOI:** http://dx.doi.org/10.7554/eLife.01071.005

**Figure 1—figure supplement 3.**
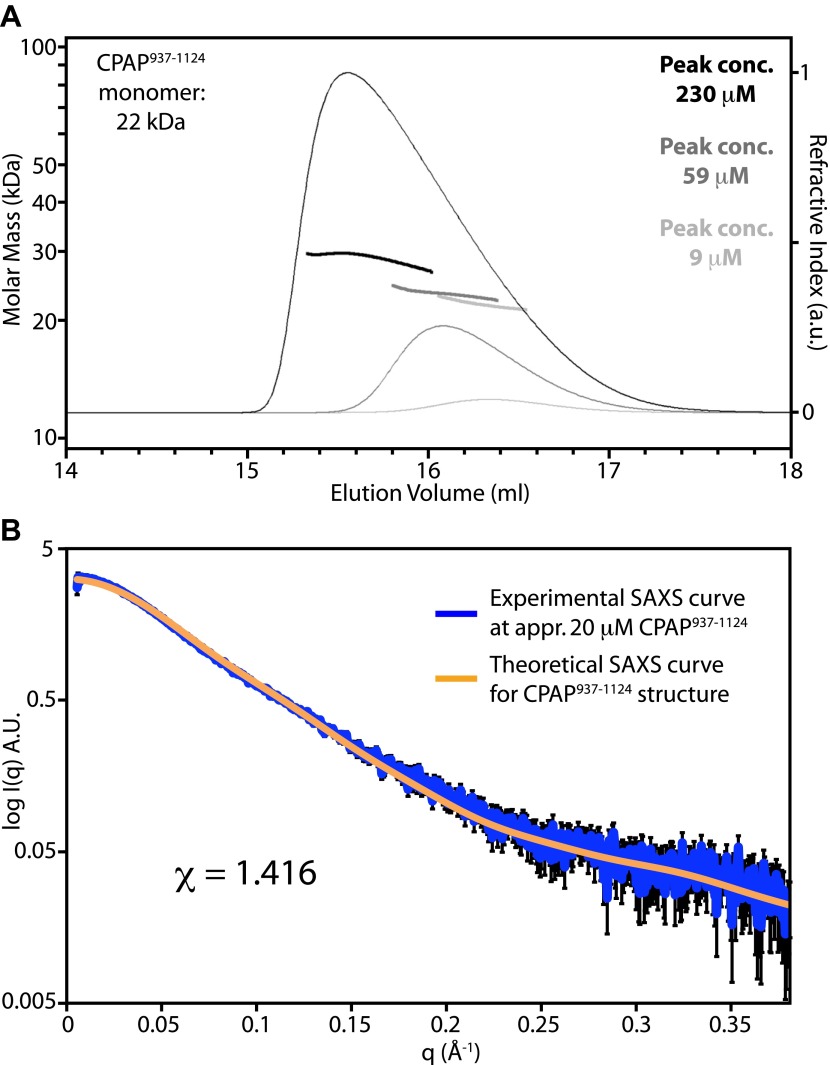
The TCP domain of CPAP is predominantly monomeric in solution. (**A**) Panel showing size exclusion chromatography coupled to multi-angle light scattering (SEC-MALS) chromatograms. CPAP^937–1124^ was injected at concentrations of approximately 70 μM (light grey), 460 μM (dark grey) and 2.4 mM (black). The corresponding chromatogram traces (thin lines) show the refractive index signal. The concentrations of the CPAP^937–1124^ monomer measured at the peaks are indicated, and heavy solid lines across the peaks show the calculated molar masses. When averaged across the central 50% of the peaks, these molar masses were 22 kDa, 23 kDa, and 29 kDa, respectively. The theoretical molecular weight of a CPAP^937–1124^ monomer is 22 kDa. The Rh of CPAP^937–1124^ determined at the intermediate concentration was 2.9 ± 0.15 nm, which is significantly larger than expected for a globular protein of this mass and thus is consistent with the extended crystallographic structure. SEC-MALS measurements were performed using a Wyatt Heleos II 18 angle light scattering instrument coupled to a Wyatt Optilab rEX online refractive index detector. Detector 12 in the Heleos instrument was replaced with Wyatt's QELS detector for dynamic light scattering measurement. Samples (100 μl) were resolved on a Superdex S-200 10/300 analytical gel filtration column (GE Healthcare, Little Chalfont, UK) running at 0.5 ml/min in 25 mM bis Tris pH 7.2, 100 mM NaCl buffer before passing through the light scattering and refractive index detectors in a standard SEC-MALS format. Protein concentration was determined from the excess differential refractive index based on 0.186 RI increment for 1 g/ml protein solution. The concentration and the observed scattered intensity at each point in the chromatograms were used to calculate the absolute molecular mass from the intercept of the Debye plot using Zimm's model as implemented in Wyatt's ASTRA software. Autocorrelation analysis of data from the dynamic light scattering detector was also performed using Wyatt's ASTRA software, and the translational diffusion coefficients determined were used to calculate the hydrodynamic radius using the Stokes-Einstein equation and the measured solvent viscosity of 9.3 e-3 Poise. (**B**) Small-angle X-ray scattering (SAXS) experiment with approximately 20 μM *D. rerio* CPAP^937–1124^ in 25 mM bis Tris pH 7.2, 100 mM NaCl, 2 mM DTT. Shown in blue is the experimentally measured SAXS curve of CPAP^937–1124^ with the experimental error indicated by black bars. The orange line shows the fitted theoretical SAXS curve of CPAP^937–1124^ derived from its crystal structure. Fitting was done using CRYSOL (Svergun et al., 1995) and resulted in a χ-value of 1.416. A.U., arbitrary units. At higher CPAP^937–1124^ concentrations the fit became less good due to the tendency of CPAP^937–1124^ to self-associate at these concentrations as revealed by a gradual increase of the derived Rg values. SAXS data were collected at the European Synchrotron Radiation Facility (ESRF), Grenoble, France, at beamline ID14–3. Measurements were done at 10°C at a wavelength of 0.931 Å with the standard beamline settings using a PILATUS 1M detector (Dectris, Baden, Switzerland). To minimise radiation damage, a flow cell was used for the measurements. Collected data was buffer subtracted using PRIMUS (Konarev et al., 2003) and the beamstop shadow removed by cutting the data at a q-value of 0.055 nm^−1^. Above a q-value of 3.8 nm^−1^ the data became too noisy to be interpretable and the data were therefore cut at this value. **DOI:** http://dx.doi.org/10.7554/eLife.01071.006

**Figure 1—figure supplement 4.**
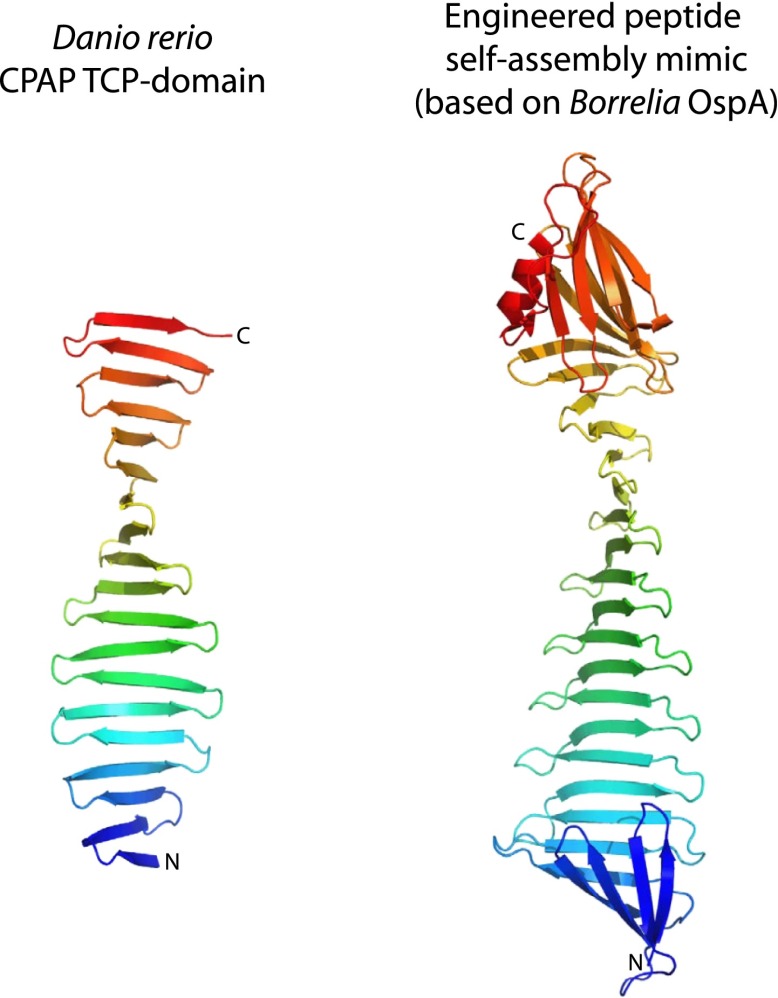
The TCP domain resembles engineered peptide-assembly mimics used to study β-rich self-assemblies. Side-by-side comparison of the apo-structure of the *D. rerio* TCP-domain of CPAP (left) with the structure of an engineered peptide-assembly mimic based on *Borrelia* OspA (right) that is used to study β-rich self-assemblies (PDB code 2FKJ, chain A). Structures are shown as ribbon presentations and are rainbow-coloured from N- to C-terminus. Note that the conformation of the peptide-self-assembly mimic is maintained by two globular domains capping both ends of its β-sheet. In contrast, the TCP domain entirely lacks a hydrophobic core. **DOI:** http://dx.doi.org/10.7554/eLife.01071.007

**Figure 1—figure supplement 5.**
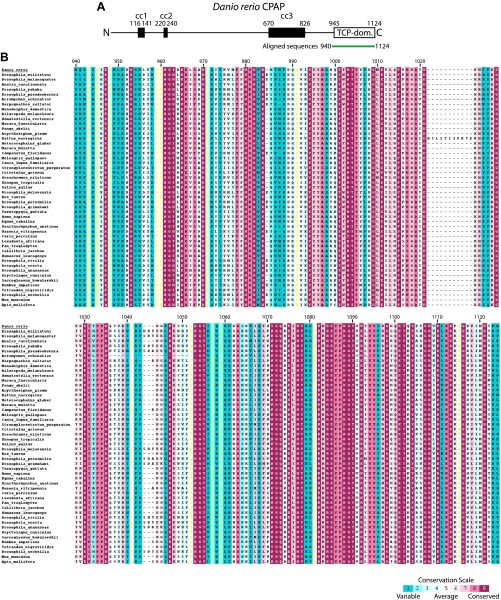
Multiple sequence alignment of the TCP domain of CPAP. The numbering refers to *D. rerio* CPAP. The alignment is coloured by conservation according to the Consurf conservation score from cyan (variable) to burgundy (conserved). **DOI:** http://dx.doi.org/10.7554/eLife.01071.008

**Table 1.** Characterisation of the CPAP:STIL interaction in vitro **DOI:** http://dx.doi.org/10.7554/eLife.01071.009

*Danio rerio* STIL peptide in syringe	*Danio rerio* CPAP^937–1124^ TCP domain in cell	Number of binding sites (N)	SD N	K_D_ (μM)	SD K_D_ (μM)	ΔH (kcal/mol)	SD (kcal/mol)	n (number of measurements)	Factor change in K_D_
STIL^404–448^	WT	1.07	0.04	1.9	0.2	−10.1	0.3	5	1
STIL^411–428^	WT	0.98	0.03	4	0.3	−11.3	0.5	3	2
STIL^429–448^	WT	0.97	0.08	540	130	−6.3	0.6	2	∼280

Binding parameters between *D. rerio* CPAP and various *D. rerio* STIL constructs obtained from ITC experiments. Fitting was performed with N as a variable. Constraining N to a fixed value of 1 during fitting produced K_D_ values that were within the experimental error of those tabulated here.

### The CPAP TCP domain adopts a unique extended open β-sheet conformation that packs against a series of conserved prolines in STIL

To understand how CPAP and STIL interact at the molecular level, we obtained the crystal structures of the TCP-domain of *D. rerio* CPAP^937–1124^, both on its own and in a complex with *D. rerio* STIL^408–428^ (Figure 1B,C,E; Table 2, Table 3). In both structures, the TCP domain adopts a nearly identical conformation, suggesting that no significant conformational change occurs in CPAP upon binding to STIL (RMSD = 1.5 Å ± 0.2 Å over 148 ± 4 Cα pairs). The TCP domain folds into a single layer β-sheet comprising ∼20 consecutive antiparallel strands connected by type I β-turns and is stabilised by an extensive hydrogen-bonding network. The resulting sheet shows a twist of approximately 13° (i.e., the angle between the consecutive, hydrogen-bonded strands), slightly lower than the average value of 20° observed for typical β-sheets (Chothia, 1973; Murzin, 1992). Individual β-hairpins correspond to previously noted (Islam et al., 1993; Hung et al., 2000) repeats in the TCP domain sequence; the turns of these hairpins are often constituted by a PDG motif explaining the high frequency of proline and glycine residues in this domain (Figure 1—figure supplement 2). Crystal packing interactions involve only small protein interfaces, suggesting that the protein is biologically active as a monomer. Indeed both small-angle X-ray scattering (SAXS) and size-exclusion chromatography—multi angle light scattering (SEC-MALS) experiments demonstrate that the TCP domain is predominantly monomeric in solution (Figure 1—figure supplement 3).

The structure of the TCP domain represents an unusual, novel architecture. It is reminiscent of the β-sheet conformation proposed to exist within amyloid fibrils and resembles engineered water-soluble peptide self-assembly mimics (PSAMs) used to study β-rich self-assemblies (Makabe et al., 2006). In contrast to these PSAM structures whose conformation is maintained by two globular domains capping both ends of the β-sheet, the TCP domain stably exists on its own. (Figure 1—figure supplement 4). The TCP domain structure lacks a defined hydrophobic core typical for globular domains, and both sides of its β-sheet are exposed to the solvent and well hydrated.

The structure of the CPAP–STIL complex revealed that the STIL peptide binds in a polyproline II helical conformation along one edge of the TCP domain β-sheet. The STIL peptide binds to CPAP by four main mechanisms (Figure 1C). First, three STIL prolines (P417, P421, and P423) pack against aromatic CPAP residues (F978, Y996, and F1015) in a way that resembles target motif recognition by other described proline-rich motif (PRM) binding domains (Kay et al., 2000). Second, R418 (STIL) makes a cation-π interaction with the phenyl ring of Y994 (CPAP). Third, STIL R418 is further involved in a water-mediated hydrogen bonding network that includes CPAP residues H1003 and T1005. Finally, sidechain–mainchain interactions are formed between CPAP residues Y994, Q1019, and E1021 and the bound STIL peptide. The CPAP and STIL residues involved in this interaction are highly conserved across metazoans (Figure 1C).

Sequence conservation of the TCP domain is not confined to this section of our structure but extends further along the same edge of the sheet (Figure 1B). This additional conserved region contains aromatic residues that are arranged similar to those that pack against the proline residues of the bound STIL peptide in our crystal structure (Figure 1B,C). Intriguingly, the C-terminal part of STIL’s CR2 region (omitted to obtain diffraction grade crystals) contains two highly conserved proline residues (P435 and P438 in *D. rerio*) that would be well positioned to bind to these aromatic residues in an analogous way (Figure 1A,B). Thus, we speculate that the entire CR2 region of STIL spanning from residue 417 to residue 438 (*D. rerio*) may be bound all along the edge of the TCP domain. Although our ITC experiments suggest that these putative additional contacts are insufficient to establish strong binding between STIL and CPAP (Figure 1D) they may contribute cooperatively to the CPAP–STIL interaction once the N-terminal proline-rich region in CR2 established binding. We conclude that the TCP domain of CPAP adopts a unique extended open β-sheet conformation that recognises a series of conserved prolines in the CR2 region of STIL.

### The CPAP E1021V MCPH mutation reduces the binding affinity of the CPAP–STIL interaction

**Figure 2.**
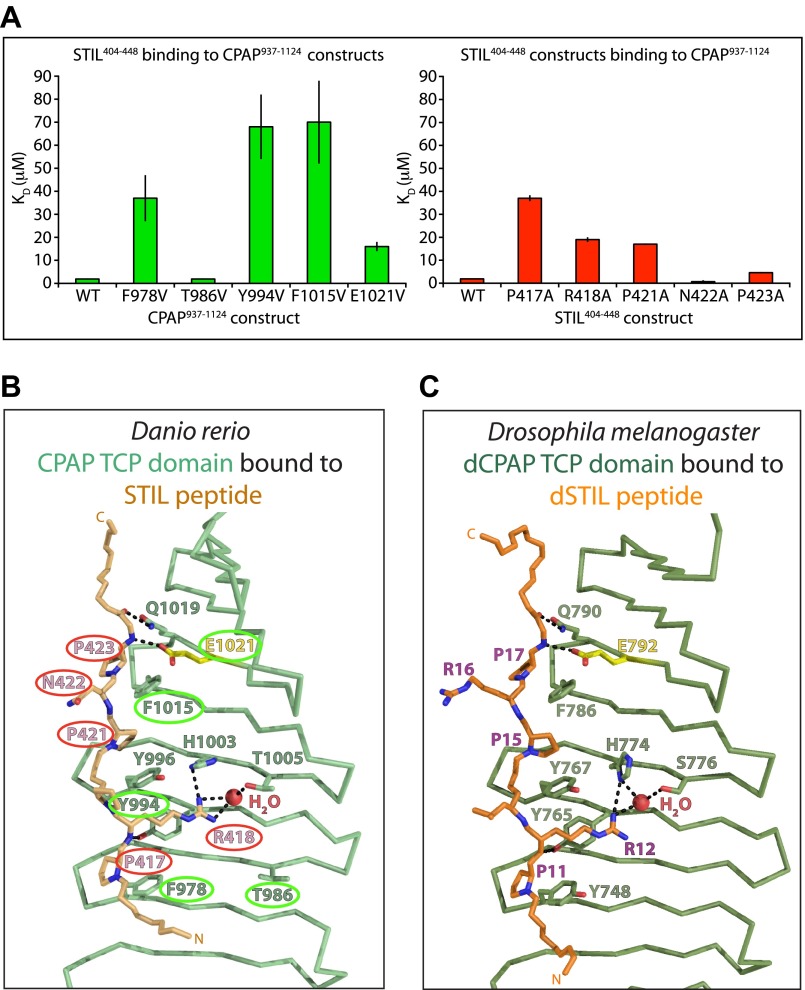
Mutational analysis of the CPAP:STIL interaction in vitro and conservation of the interaction across species. (**A**) Graphs showing the binding constants (K_D_) determined by ITC for the interaction between WT and mutant constructs of CPAP^937–1124^ and STIL^404–448^. Left panel, WT and various mutant forms of CPAP^937–1124^ binding to WT STIL^404–448^ (T986 is a non-interacting residue included as a negative control). Error bars, standard deviation. Right panel, WT and various mutant STIL^404–448^ constructs binding to WT CPAP^937–1124^ (N422 is a non-interacting residue included as a negative control). Error bars, standard deviation. The wild-type measurements are the same as shown in Figure 1D and are shown again for comparison to the mutants. (**B** and **C**) Close-up view of the CPAP (green):STIL (orange) interaction interface from *D. rerio* (**B**) and *Drosophila* (**C**). Interface residues are shown as sticks, in yellow is the Glutamate residue in *Drosophila* and *D. rerio* CPAP that is equivalent to E1235 in human CPAP (mutated in MCPH). Residues of the *D. rerio* protein mutated for ITC experiments are ringed in green (CPAP) or red (STIL). Dotted black lines indicate hydrogen-bonds. The conserved bound water molecule is shown as a red sphere. **DOI:** http://dx.doi.org/10.7554/eLife.01071.010

The involvement of CPAP E1021 in the interaction with STIL in zebrafish is potentially significant, as the equivalent residue in human CPAP (E1235) is mutated to valine in some MCPH patients. To test whether this mutation disrupts the organisation of the TCP domain, we obtained the crystal structure of *D. rerio* CPAP^937–1124^ carrying the E1021V mutation (Figure 1F; Table 2). The structure of the wild-type and the mutant TCP domain were virtually identical (RMSD = 0.1 Å over 142 Cα pairs) demonstrating that the TCP domain structure was not compromised. To test whether this mutation perturbed the interaction with STIL, we purified WT and various other mutant forms of *D. rerio* CPAP^937–1124^ in which we valine substituted residues that our crystal structure suggested to be important for binding (Figure 2B). Circular dichroism (CD) spectra indicated that the mutant forms of the TCP domain were correctly folded with a predominantly β-type profile (Figure 2—figure supplement 1). ITC experiments with WT *D. rerio* STIL^404–448^ showed that the mutation of residues F978, Y994, and F1015 decreased the binding strength by ∼20 to 40-fold (Figure 2A, left; Table 4), while mutation of E1021 decreased the binding strength by approximately eightfold. In contrast, mutation of T986, which is not predicted to be in the interaction interface, did not detectably perturb binding.

**Figure 2—figure supplement 1.**
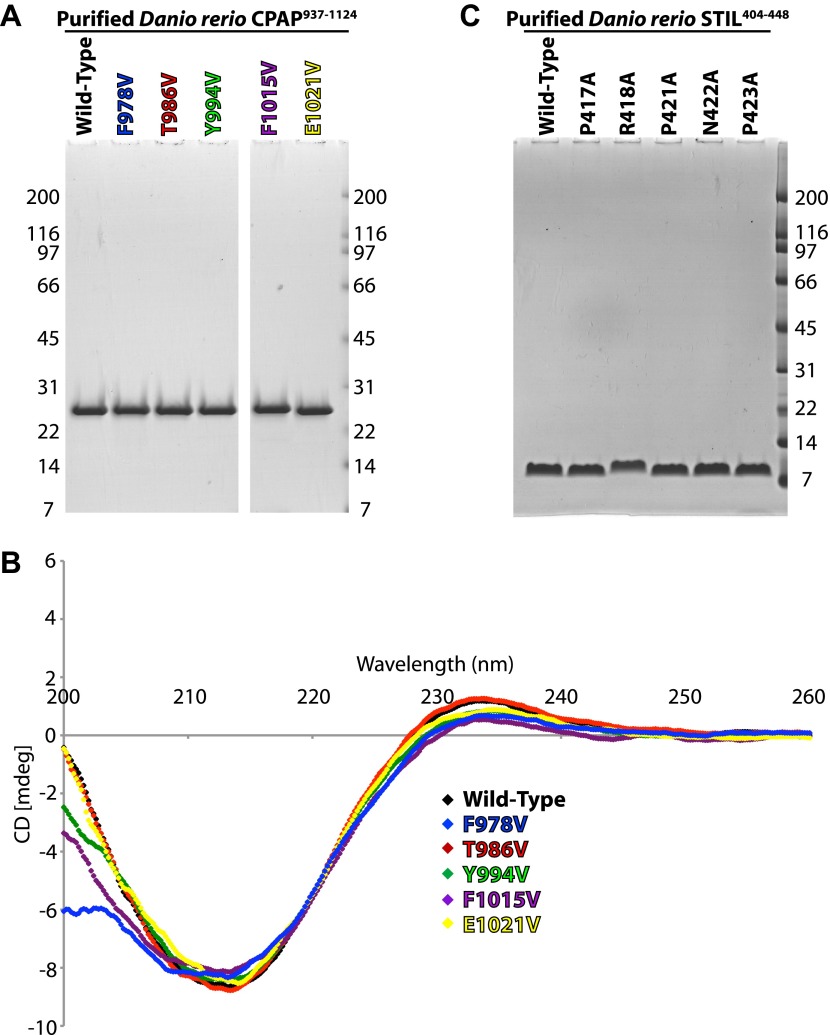
Characterisation of the *D. rerio* TCP domain mutants and STIL peptide mutants used for thermodynamic analysis. (**A**) Coomassie stained SDS-PAGE gel of purified, recombinant CPAP^937–1124^ and its mutants (as labelled in colours above the gel). (**B**) Buffer-subtracted circular dichroism (CD) spectra of *D. rerio* CPAP^937–1124^ and its mutants in 10 mM Na-Phosphate pH 7.3 at approximately 200 μg/ml. Spectra were recorded on a JASCO J-810 from 260 to 190 nm in 0.2 nm steps at 20°C and are colour-coded as in (**A**). The data were cut at 200 nm as the detector was saturated below this wavelength for some constructs as indicated by a high HT voltage. (**C**) Coomassie stained SDS-PAGE gel showing purified, recombinant *D. rerio* STIL^404–448^ and its mutants. **DOI:** http://dx.doi.org/10.7554/eLife.01071.011

We also purified mutant forms of the *D. rerio* STIL^404–448^ peptide and tested their ability to interact with the WT *D. rerio* TCP domain in ITC experiments (Figure 2A, right, Figure 2—figure supplement 1C; Table 4). Alanine substitution of P417, R418 or P421 decreased the binding strength by ∼10 to 20-fold and alanine substitution of P423 by approximately twofold to threefold. In contrast, the mutation of residue N422, which is not predicted to be in the interaction interface, did not compromise binding. Taken together these results lend strong support to our structural model and indicate that the E1021V MCPH mutation leads to roughly an order of magnitude decrease in affinity of the CPAP–STIL interaction.

### The CPAP–STIL interaction is highly conserved

The sequence conservation of the CPAP TCP domain (Figure 1—figure supplement 5) and the CR2 region of STIL (Figure 1—figure supplement 1) suggests that this interaction may be conserved. To confirm this, we solved the crystal structure of the TCP domain from *Drosophila melanogaster* DSas-4 (dCPAP) (residues 700–901) in complex with the region of Ana2 (dSTIL) equivalent to CR2 (residues 1–47) (Table 2, Table 5, Table 6; Figure 2C). The dSTIL–dCPAP interaction interface in this structure was highly similar to the *D. rerio* complex (inter-species alignments of the structures yielded an average pairwise RMSD of 1.2 ± 0.2 Å across an average of 118 ± 4 Cα pairs). Indeed, all copies of the complex obtained in the structures from both species superimposed well and exhibited the same four major groups of binding interactions as described for the *D. rerio* structure. This conservation includes the contact made by the E792 residue in dCPAP (the equivalent of the E1235 residue in human CPAP that is mutated in MCPH). Together, these data allow us to determine a consensus CPAP binding motif in metazoan STIL proteins (PRxxPxP, Figure 1—figure supplement 1) and suggest that the described CPAP–STIL interaction constitutes a highly conserved step in centriole biogenesis.

### The CPAP–STIL interaction is essential for centriole assembly in vivo

**Figure 3.**
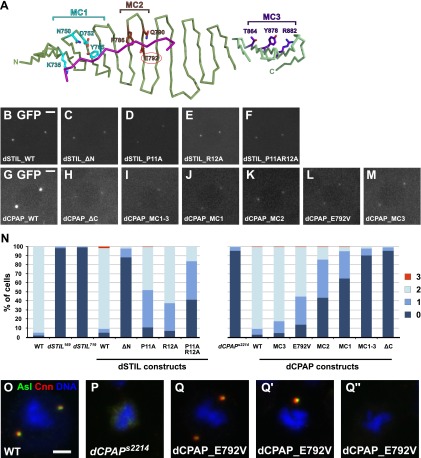
The interaction between dCPAP and dSTIL is essential for centriole duplication in *Drosophila*. (**A**) Schematic view of the complex between dCPAP (green) and dSTIL (magenta) with the residues mutated in MC1 (cyan), MC2 (brown) and MC3 (dark purple) indicated as coloured sticks. The MCPH residue E792 is circled in red. Note that MC1 and MC2 are mapped onto the *Drosophila* structure (dark-green backbone), while MC3 had to be mapped onto the backbone of the *D. rerio* structure (light green backbone). Although highly conserved between *Drosophila* and *D. rerio* (Figure 1—figure supplement 5) this region was not visible in the electron density map of the *Drosophila* structure probably due to its partial unfolding to enable packing interactions within the crystal. (**B**–**M)** Panels show representative still images taken from movies of *Drosophila* embryos expressing the indicated dCPAP-GFP or dSTIL-GFP constructs. Note that all analyses were performed in the presence of endogenous WT dCPAP or dSTIL, and that all images were acquired with the same microscope settings at the same stage of the cell cycle. (**B**–**F**) dSTIL-GFP constructs localise to centrosomes at similar levels. (**G**–**M**) All mutant dCPAP-GFP constructs localise to centrosomes, but at strongly reduced levels compared to wild-type dCPAP-GFP. (**N**) Graphs show the percentage of cells with 0, 1, 2, and 3 centrosomes in the genotypes analysed (as indicated). All dSTIL-GFP and dCPAP-GFP constructs were analysed in their respective mutant backgrounds. Note that this experiment was performed blind. (**O**–**Q**′′) Panels show third instar larval brain cells of various genotypes in metaphase. Cells were stained for the centriolar protein Asterless (Asl—green) and the PCM component Centrosomin (Cnn—red) and DNA (blue). Wild-type metaphase cells have two centrosomes (**O**), whereas centrosomes are mostly absent in third instar larval brain cells from *dCPAP* mutants (**P**). As an example, representative images of *dCPAP* mutant cells expressing the dCPAP_E792V-GFP construct are shown that were scored with 2 (**Q**), 1 (**Q**′) or no (**Q**′′) centrosomes. Scale bars = 3 μm. **DOI:** http://dx.doi.org/10.7554/eLife.01071.012

Since the binding mechanism of CPAP and STIL is conserved between zebrafish and *Drosophila*, we turned to *D. melanogaster* as a model system to address the functional relevance of this interaction in vivo. In flies, the lack of dCPAP or dSTIL leads to centriole loss and a consequent severe uncoordinated (unc) phenotype due to the lack of basal bodies and so cilia in Type I sensory neurons. These flies lack all mechano- and chemo-sensation and, although viable, they usually die shortly after eclosion, as they cannot feed or move in a coordinated fashion (Kernan et al., 1994; Basto et al., 2006; Wang et al., 2011). We examined the ability of various GFP-tagged versions of dCPAP and dSTIL to rescue the centriole loss observed in these mutants and assayed their ability to localise to centrosomes in the presence of endogenous dCPAP or dSTIL (Figure 3).

**Figure 3—figure supplement 1.**
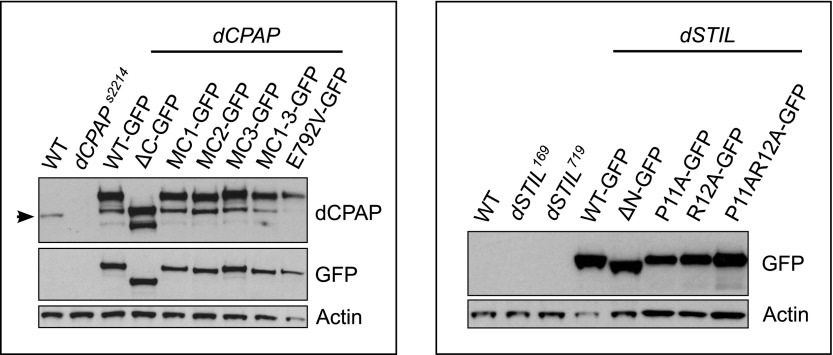
Protein expression levels of GFP-tagged dCPAP and dSTIL constructs in *dCPAP* or *dSTIL* mutant *Drosophila* brain cells and quantification of their centriole/centrosome numbers. Panels show western blots of third instar larval brain samples probed with antibodies against GFP (recognising the fusion proteins), actin (as a loading control) and dCPAP (recognising both the fusion proteins and the endogenous protein) (highlighted by an arrow). All fusion proteins were expressed in their respective mutant backgrounds, as indicated by the black lines. Left panel, the dCPAP fusion proteins were expressed at approximately equal levels (when compared to the actin control) but were all moderately overexpressed compared to the endogenous dCPAP protein. Right panel, dSTIL_WT, dSTILΔN and dSTILP11AR12A were expressed at slightly higher levels than dSTIL_P11A and dSTIL_R12A, and all GFP fusion proteins were strongly overexpressed when compared to endogenous dSTIL (data not shown). **DOI:** http://dx.doi.org/10.7554/eLife.01071.013

We first expressed a version of dSTIL that lacks the first 45 aa (including the PRxxPxP motif required for the interaction with dCPAP). GFP-tagged wild-type dSTIL (dSTIL_WT-GFP) served as a control. Both proteins were expressed at similar levels and localised strongly to centrosomes in the presence of endogenous dSTIL (Figure 3B,C; Figure 3—figure supplement 1). Only the wild-type version, however, was able to rescue the unc phenotype and the centriole loss phenotype of the *dSTIL* mutant (Figure 3N; Table 7). To further characterise the dCPAP binding domain in vivo we mutated the first proline and arginine of the PRxxPxP motif of dSTIL to alanine, both separately and in combination (P11A, R12A, and P11A:R12A, Figure 2C). All three constructs strongly localised to centrosomes in the presence of endogenous dSTIL (Figure 3D–F, Figure 3—figure supplement 1). Both single mutants rescued the unc phenotype of the *dSTIL* mutation while the double mutant failed to do so (data not shown). The single mutants P11A and R12A were also able to partially rescue the centriole loss phenotype, whereas the double mutant P11A:R12A showed only a poor rescue (Figure 3N; Table 7). These data strongly suggest that the interaction with dCPAP is essential for dSTIL function in centriole assembly.

We next deleted the entire TCP-domain of dCPAP (dCPAP_ΔC), or expressed GFP fusion proteins carrying mutation clusters (MCs) altering 3–4 residues in different regions of the TCP domain (Figure 3A). Mutation clusters were designed that targeted central (dCPAP_MC1) or peripheral (dCPAP_MC2) residues in the dSTIL binding domain, as well as residues that are predicted to not significantly be involved in complex formation (dCPAP_MC3), according to the crystal structure and the ITC data (Figure 3A, Figure 2A, Figure 1D; Table 1). We also analysed dCPAP_E792V-GFP lines, which carried the MCPH equivalent mutation E792V (E1235V in humans and E1021V in zebrafish CPAP). All transgenic dCPAP-GFP proteins were expressed at approximately equivalent levels in vivo, but were moderately overexpressed compared to endogenous dCPAP (Figure 3—figure supplement 1). Wild-type dCPAP-GFP localised strongly to centrosomes and rescued both the unc phenotype and the centriole loss phenotype (Figure 3G,N; Table 7). Strikingly, the rescuing ability of the mutant constructs strongly correlated with the predicted strength of dSTIL binding. dCPAP_ΔC-GFP failed to rescue, dCPAP-MC1 and dCPAP-MC2 rescued poorly, the MCPH mutation E792V showed an intermediate phenotype, while dCPAP-MC3 exhibited a robust rescue (Figure 3N; Table 7). Interestingly, when compared to wild-type dCPAP-GFP, all mutant constructs (including dCPAP_MC3) localised only weakly to centrosomes (Figure 3H–M). Together, these data suggest that the interaction between dCPAP and dSTIL is a key step in centriole assembly and is essential for centriole duplication. Furthermore, they indicate that low total levels of dCPAP at centrosomes might be sufficient for centriole duplication, as long as some interaction with dSTIL is maintained.

### The TCP domain of *C. elegans* SAS-4 is required for its interaction with SAS-5 and for centriole assembly

**Figure 4.**
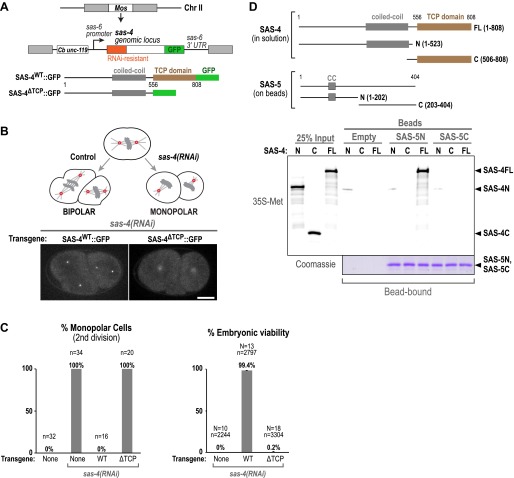
The TCP domain of *C. elegans* SAS-4 is required for its interaction with SAS-5, its localisation to centrioles, and for centriole assembly. (**A**) Schematic illustration of the MosSCI system used for generating single-copy *sas-4* transgene insertions. (**B**) A schematic illustration of the monopolar spindle assay for centriole duplication in *C. elegans* embryos. Panels show maximum intensity projections of representative fluorescence confocal z-series taken of *sas-4(RNAi)* embryos expressing either WT or ΔTCP SAS-4::GFP. Transgenic SAS-4^WT^::GFP localises to sharp foci representing the centrioles, whereas SAS-4^ΔTCP^::GFP localises diffusely to the pericentriolar material. Bar, 10 μM. (**C**) Graphs show the quantification of second division monopolar spindles (left) and embryonic viability (right) after *sas-4(RNAi)* and rescue with either a WT or ΔTCP *sas-4::gfp* transgene. (**D**) Panels show autoradiographs (top panel) and a Coomassie stained gel from a Ni-NTA pull-down experiment with ^35^S-labelled in vitro translated SAS-4 fragments (prey) and SAS-5-6xHis fragments (baits). **DOI:** http://dx.doi.org/10.7554/eLife.01071.014

It has been proposed that SAS-5 is the *C. elegans* homolog of the STIL proteins in flies and vertebrates, but there is little sequence homology between these proteins (Stevens et al., 2010a). We failed to identify an unambiguous PRxxPxP motif in worm SAS-5, so we tested whether the TCP domain of SAS-4 (the *C. elegans* CPAP homologue) is functionally important. We used the Mos single-copy insertion system (MosSCI; Frøkjær-Jensen et al., 2008) to generate transgenic lines with single-copy transgenes under the control of *sas-6* regulatory sequences integrated at a specific site on chromosome II (Figure 4A). Transgenes were generated expressing GFP fusions with either WT SAS-4 (SAS-4^WT^::GFP) or a form in which the C-terminal TCP domain (aa 557–808) had been deleted (SAS-4^ΔTCP^::GFP); both transgenes contained a 497 bp resequenced region in their N-terminal coding region (preserving codon usage) that rendered them resistant to RNAi-mediated depletion (Figure 4A).

SAS-4 depletion by RNAi prevents centriole assembly, resulting in a signature phenotype characterised by a normal first mitotic division followed by monopolar spindles during the second division (Figure 4B,C; O’Connell et al., 2001). This phenotype arises because the sperm that fertilise the SAS-4-depleted oocytes carry two normal centrioles, since sperm are produced prior to introduction of the dsRNA. The two sperm-derived centrioles organise two centrosomes, and the first mitotic division appears normal. If no new centrioles form, each daughter cell inherits a single sperm-derived centriole leading to monopolar spindles in the second division and 100% embryonic lethality (Figure 4C). Both the monopolar spindle phenotype and embryonic viability were fully rescued by the WT *sas-4::gfp* transgene (aa 1–808), but not by the ΔTCP transgene (aa 1–556) (Figure 4B,C). While SAS-4^WT^::GFP targeted to centrioles in the absence of the endogenous protein, SAS-4^ΔTCP^::GFP did not, and instead exhibited a diffuse accumulation in the pericentriolar material (Figure 4B). Thus, the SAS-4 TCP domain is required for SAS-4 to accumulate at centrioles and become incorporated into the microtubule-containing outer centriole wall.

To determine if the failure of SAS-4^ΔTCP^::GFP to become incorporated in the centriole outer wall could be due to an inability to interact with SAS-5, we performed a pull-down assay to determine whether ^35^S-labelled in vitro translated SAS-4 fragments could interact with the N-terminal or C-terminal regions of SAS-5 bound to beads (Figure 4D). In vitro translated full-length SAS-4 interacted specifically with the N-terminal domain (aa 1–202) of SAS-5. Interestingly, we could not further narrow down the region of SAS-4 required for this interaction. Neither the SAS-4 N-terminal nor C-terminal region (which includes the TCP domain) alone could be pulled down by SAS-5. This result suggests that although the TCP domain is required for SAS-4 to interact with SAS-5, it is not sufficient. Together, these data suggest that a TCP domain-dependent interaction between SAS-4/CPAP and SAS-5/STIL is conserved and essential for centriole duplication in *C. elegans*, but that the precise interaction interface may have diverged.

## Discussion

Only a small set of conserved centriolar proteins is essential for centriole assembly (Brito et al., 2012; Gonczy, 2012) and some of these proteins, like CPAP and STIL, have been linked to microcephaly in humans (Leal et al., 2003; Bond et al., 2005; Gul et al., 2006; Thornton and Woods, 2009; Darvish et al., 2010). However, there is currently little structural understanding on how these proteins interact with one another, how mutations in them cause microcephaly in humans and how these interactions are regulated.

Here we have solved the crystal structures of the CPAP–STIL complex from zebrafish and *Drosophila*. We showed that the CPAP TCP domain folds into an elongated open-sided β-meander that consists of ∼20 consecutive antiparallel β-strands connected by type I β-turns. β-meanders are frequently found in β-barrels, β-propellers and some α+β proteins. However, what, to our knowledge, makes the TCP domain structure unique amongst naturally occurring proteins is that it solely consists of a freestanding meander β-sheet that entirely lacks a defined hydrophobic core and is not flanked by other globular domains that pack against it. We show that the TCP domain is predominantly monomeric in solution and self-interacts in its crystallised form only through small interfaces that are not conserved. Thus, despite some reminiscence to β-sheets observed in amyloid fibrils it is unlikely that the TCP domain self-associates in a similar manner.

**Figure 5.**
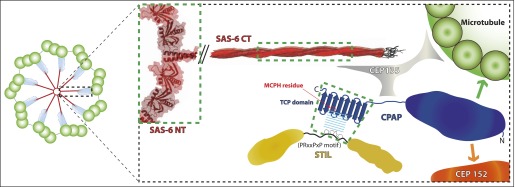
A schematic representation of protein interactions within the inner region of the centriole. In this illustration, interactions whose crystal structure have been determined are highlighted by green boxes—all other interactions are inferred from biochemical and genetic studies and so are depicted in cartoon form. The cartwheel central hub comprises SAS-6 (red) (Nakazawa et al., 2007; Kitagawa et al., 2011b; van Breugel et al., 2011). The spokes extending outward from the hub consist of a homodimeric SAS-6 coiled-coil, which extends (van Breugel et al., 2011) into a region known as the ‘pinhead’ (cyan in low magnification view, left), where CEP135 (grey) may act as a linker between SAS-6, CPAP and microtubules (Hiraki et al., 2007; Roque et al., 2012; Lin et al., 2013). CPAP (dark blue) localises more towards the periphery of the centriole (Mennella et al., 2012; Sonnen et al., 2012; Lukinavičius et al., 2013), where its N-terminal part may interact directly with both Asterless/CEP152 (Cizmecioglu et al., 2010; Dzhindzhev et al., 2010) (orange arrow) and microtubules (Hsu et al., 2008) (green arrow). In contrast STIL (yellow) localises more towards the interior of centrioles (Arquint et al., 2012), and appears to function upstream of CPAP in centriole biogenesis (Tang et al., 2011; Vulprecht et al., 2012). Thus, we propose that the C-terminal TCP domain of CPAP interacts with the conserved region 2 (CR2) of STIL towards the interior of the centriole and that this interaction is crucial for CPAP/STIL function at centrioles. The orientation of STIL in centrioles is unknown. **DOI:** http://dx.doi.org/10.7554/eLife.01071.015

Instead, we demonstrate that the TCP domain of CPAP constitutes a novel proline-rich-motif (PRM) recognition-domain that specifically binds to a short target motif in STIL. Although the overall sequence identity of the CPAP and STIL proteins between *Drosophila* and zebrafish is relatively low (∼22% and ∼13%, respectively), our structural analysis revealed that the interaction interface is conserved, confirming the previous proposal that fly Ana2 is the functional homologue of vertebrate STIL (Stevens et al., 2010a). Our characterisation of the binding interface also allowed us to define a consensus-binding site (PRxxPxP) for the CPAP TCP domain in STIL that is conserved across metazoa. Our mutational analysis of the interface demonstrates a remarkable correlation between the ability of mutant proteins to bind to one another in vitro and their ability to support centriole assembly in vivo, providing compelling support for our structural model of the metazoan CPAP–STIL complex. These data strongly suggest that the interaction between CPAP and STIL is a conserved, essential step in centriole biogenesis. A schematic model that places this interaction in the context of a possible centriole assembly pathway is shown in Figure 5.

The high degree of sequence divergence between vertebrate STIL, *Drosophila* Ana2 and *C. elegans* SAS-5 suggests that STIL homologs are under particularly strong lineage-specific selection. Despite the many sequence changes between *Drosophila* Ana2 and vertebrate STIL, our work suggests that the interaction interface between Ana2/STIL and dSAS-4/CPAP TCP domain has been retained, highlighting its importance. Even in *C. elegans*, which is the most divergent of the functionally characterised STIL homologs, our work indicates that the SAS-4 TCP domain is essential for centriole assembly, and that a TCP-domain dependent interaction between SAS-4 and SAS-5 has been conserved. Nevertheless, as the SAS-4 TCP domain is not sufficient for interaction with SAS-5 and we were unable to identify a PRxxPxP interaction motif in worm SAS-5, more work will be needed to understand the SAS-4—SAS-5 interaction in *C. elegans* and its relationship to the CPAP–STIL interaction in other metazoans.

A surprising aspect of our findings is that the E792V (MCPH) mutant and all three of the mutation clusters (MCs) that we analysed in dCPAP localise poorly to centrosomes. For the E792, MC1, and MC2 mutations this could be expected, as these are all predicted to perturb the interaction between dCPAP and dSTIL (as is the case with similar mutations in zebrafish CPAP in our in vitro binding assays), and this would be predicted to perturb the recruitment of dCPAP to centrioles. The MC3 cluster, however, is not predicted to lie in a strong interaction interface and, unlike the MC1 and MC2 mutation clusters, it can rescue the centriole duplication defect in dCPAP mutant cells nearly as efficiently as the WT protein. Possibly, an interaction with another protein that plays some part in recruiting dCPAP to centrioles might be perturbed by these mutations. Alternatively, similar to the situation with *C. elegans* SAS-4 (Dammermann et al., 2008), dCPAP may localise to both centrioles and the PCM. It might therefore be PCM and not centriole recruitment that is affected by the mutation clusters. If this were the case it would be hard to discern an additional partial loss of centriole recruitment, as this loss would be masked by the PCM pool of dCPAP, especially under conditions of moderate overexpression of dCPAP. Importantly, however, our findings demonstrate that even very reduced amounts of centrosomal dCPAP can support robust centriole duplication as long as this protein can interact efficiently with dSTIL.

Our studies provide the first structural insight into the nature of the link between centrioles and human microcephaly. It is unclear why mutations in genes encoding key centriole or centrosome proteins can lead to such a specific neuro-developmental disorder in humans. It is widely assumed that some aspect of centriole/centrosome function must be particularly important in human neural progenitor cells, and that the failure of these cells to proliferate in an appropriate manner underlies the small brain size in affected individuals (Megraw et al., 2011). One possibility, based on the fact that these neural progenitors seem to divide asymmetrically (Siller and Doe, 2009; Megraw et al., 2011), is that centrioles/centrosomes may play a particularly important role in properly orienting the spindle during asymmetric divisions, and division orientation could in turn be required for the maintenance of neuronal progenitors. This appears to be the case in flies, where mutations in dCPAP/DSas-4 and dSTIL/Ana2 lead to defects in the asymmetric division of the neural stem/progenitor cells (Basto et al., 2006; Wang et al., 2011). However, there are other possible explanations. Human neural progenitor cells form primary cilia, for example, and signalling through the cilium could be perturbed if centriole assembly is perturbed (Han and Alvarez-Buylla, 2010; Megraw et al., 2011). Moreover, several studies have linked centriole and centrosome malfunction to defects in DNA damage repair (DDR) pathways (Megraw et al., 2011), and mutations in MCPH genes can also lead to more severe phenotypes in humans that may be related to DDR pathway malfunction (Al-Dosari et al., 2010; Kalay et al., 2011; Megraw et al., 2011).

A previous analysis of the behaviour of various CPAP mutant proteins (modelled on MCPH mutations) in human cells revealed some surprising findings (Kitagawa et al., 2011a). The deletion of the TCP domain or the mutation of E1235 to Valine did not effect the localisation of CPAP to the centriole, although centriole duplication was compromised by both mutations. Moreover, overexpression of the E1235V mutant protein was able to promote centriole overgrowth to a greater extent than the WT protein, suggesting that it may have acquired some enhanced functionality. The structures we report here reveal that E1235 is one of the several residues involved in the binding interface with STIL, making an important sidechain–mainchain contact. This structural model explains how the E1235V mutation can compromise complex formation, and we have confirmed that this is the case with zebrafish proteins in vitro. Moreover, the equivalent mutation in flies leads to inefficient centriole assembly, but this process is not abolished. Taken together, our data strongly suggest that it is a partial failure in centriole assembly that is the primary cause of microcephaly in these patients. The challenge now is to understand how inefficient centriole assembly leads to microcephaly in humans.

## Materials and methods

### Recombinant protein expression and purification

*D. rerio* CPAP^937–1124^ was cloned from *D. rerio* cDNA. Proteins were expressed in *Escherichia coli* BL21 (DE3) Rosetta as N-terminally His-tagged constructs, and purified via immobilised metal ion affinity chromatography (NiNTA; Qiagen, Hilden, Germany), proteolytic tag cleavage, followed (optionally) by size-exclusion chromatography and ion-exchange chromatography using standard methods. The selenomethionine derivative protein was expressed in selenomethionine supplemented M9 medium and purified in the same way. Purified constructs contained the sequence GPHM at the N-termini that stem from the cloning and protease cleavage sites.

*D. rerio* STIL^404–448^ was cloned from IMAGE clone 7147918 and expressed in *E. coli* C41 BL21 (DE3), fused to two His-tagged lipoyl domains from *Bacillus stearothermophilus* dihydrolipoamide acetyltransferase at both the N- and C terminus. The peptide was purified via NiNTA chromatography, proteolytic cleavage of the His-lipoyl domains, and ion-exchange chromatography. The purified constructs contained a G (GG for *D. rerio* STIL^404–448^ and its point-mutants) at their N-terminus and the sequence EFGENLYFQ (ENLYFQ for *D. rerio* STIL^408–428^ and *D. rerio* STIL^411–428^) at their C-terminus. These extra sequences stem from the cloning and protease cleavage sites.

Mutations of the *D. rerio* constructs were introduced into the expression vectors by site-directed mutagenesis.

Codon-optimised (GeneArt, Carlsbad, CA) *Drosophila* dSTIL^1–47^ was genetically fused to the N-terminus of *Drosophila* dCPAP^700–901^ via 3-way ligation. The fusion protein was expressed in *E. coli* B834 (DE3) as an N-terminally His-tagged fusion, and purified via NiNTA chromatography, proteolytic tag cleavage and size exclusion chromatography. The selenomethionine derivative protein was expressed using SelenoMethionine Medium (Molecular Dimensions, Newmarket, UK) and purified in the same way.

**Table 2.** Native dataset analysis and refinement statistics **DOI:** http://dx.doi.org/10.7554/eLife.01071.016

	*D. rerio* CPAP^937–1124^ WT	*D. rerio* CPAP^937–1124^ E1021V	*D. rerio* CPAP^937–1124^ + *D. rerio* STIL^408–428^ complex	*D. melanogaster* dSTIL^1–47^ − dCPAP^700–901^ fusion complex
Beamline	Diamond I02	MRC-LMB Cambridge UK	Diamond I04	Diamond I04
Space group	P21	P21	P21	P1
Wavelength (Å)	0.9786	1.5418	0.9795	0.9795
Monomers in the asymmetric unit	1	1	2	3
Unit cell dimensions (Å)	a = 52.34; b = 36.44; c = 56.44; α = 90.00; β = 117.31; γ = 90.00	a = 52.12; b = 36.48; c = 56.46; α = 90.00; β = 117.47; γ = 90.00	a = 60.25; b = 67.47; c = 61.65; α = 90.00; β = 113.92; γ = 90	a = 58.64; b = 69.91; c = 69.98; α = 86.96; β = 88.64; γ = 67.69
Resolution (Å)	29.48–1.7	36.48–1.9	56.35–2.7/2.2 (anisotropy)	64.60–2.57
Completeness(overall/inner/outer shell)	99.7/99.4/100	100/99.6/100	99.9/99.5/99.9	97.6/93.6/97.2
Rmerge (overall/inner/outer shell)	0.074/0.030/0.929	0.096/0.028/1.093	0.101/0.053/1.008	0.091/0.069/0.512
Rpim (overall/inner/outer shell)	0.029/0.012/0.369	0.039/0.012/0.456	0.050/0.027/0.505	0.061/0.035/0.449
Mean I/σI (overall/inner/outer shell)	14.6/39.9/2.0	13.8/43.4/1.8	7.6/19.0/1.4	7.6/16.3/1.7
Multiplicity (overall/inner/outer shell)	7.2/7.0/7.3	6.8/6.6/6.6	4.8/4.7/4.9	3.1/2.9/3.1
Number of reflections	19,941	14,349	21,892	31,911
Number of atoms	1595	1515	3176	3924
Waters	190	114	54	65
Rwork/Rfree (% data used)	19.9/24.4 (5.1%)	20.9/26.7 (5.0%)	23.4/27.7 (5.0%)	24.5/26.3 (5.05%)
rmsd from ideal values: bond length/angles	0.011/1.478	0.009/1.310	0.015/1.619	0.007/0.900
Mean B value	26.52	31.553	59.69	70.80
Correlation coefficient Fo-Fc/Fo-Fc free	0.961/0.942	0.955/0.926	0.954/0.933	0.854/0.836
Molprobity Score	0.97 (100^th^ percentile)	1.2 (99^th^ percentile)	1.70 (96^th^ percentile)	1.40 (100^th^ percentile)

**Table 3.** SeMet *D. rerio* CPAP^937–1124^ dataset analysis and phasing statistics **DOI:** http://dx.doi.org/10.7554/eLife.01071.017

Beamline	ESRF ID 23–1		
Space group	P21		
Wavelength (Å)	0.9791 (Peak)	0.9794 (Inflection)	0.9393 (Remote)
Unit cell dimensions (Å)	a = 52.39 b = 36.53 c = 56.34 α = 90.00 β = 117.28 γ = 90.00	a = 52.59 b = 36.60 c = 56.48 α = 90.00 β = 117.24 γ = 90.00	a = 52.49 b = 36.55 c = 56.38 α = 90.00 β = 117.26 γ = 90.00
Resolution (Å)	36.56–1.7	36.56–1.7	36.56–1.7
Completeness (overall/inner/outer shell)	100.0/99.7/100.0	100/99.2/100	100/99.7/100
Rmerge (overall/inner/outer shell)	0.09/0.048/1.296	0.127/0.047/2.840	0.092/0.046/1.370
Rpim (overall/inner/outer shell)	0.042/0.031/0.552	0.056/0.028/1.201	0.041/0.026/0.580
Mean I/sd(I) (overall/inner/outer shell)	10.7/26.0/1.5	8.9/26.4/0.7	10.8/27.4/1.4
Multiplicity (overall/inner/outer shell)	7.2/7.0/7.3	7.2/6.9/7.3	7.2/7.0/7.3
Se sites found/expected	5/7		
Overall FOM	0.306

**Table 4.** Characterisation of the CPAP:STIL interaction in vitro **DOI:** http://dx.doi.org/10.7554/eLife.01071.018

*Danio rerio* STIL^404–448^ peptide in syringe	*Danio rerio* CPAP^937–1124^ TCP domain in cell	Number of binding sites (N)	SD N	K_D_ (μM)	SD K_D_ (μM)	ΔH (kcal/mol)	SD (kcal/mol)	n (number of measurements)	Factor change in K_D_
WT	WT	1.07	0.04	1.9	0.2	−10.1	0.3	5	1
WT	F978V	0.70	0.09	37	10	−23	5	4	20
WT	T986V	1.01	0.07	1.9	0.2	−10.6	0.4	3	1
WT	Y994V	1.00	0.33	68	14	−9.5	3.8	5	36
WT	F1015V	0.93	0.13	70	18	−10.4	2.7	3	37
WT	E1021V	0.91	0.13	16	2	−8.1	0.6	3	8
WT	WT	1.07	0.04	1.9	0.2	−10.1	0.3	5	1
P417A	WT	1.06	0.02	37	1.3	−11.5	0.2	3	20
R418A	WT	1.12	0.02	19	1	−8.8	0.1	4	10
P421A	WT	1.16	0.03	17	0.3	−9.8	0.2	4	9
N422A	WT	1.09	0.03	0.7	0.05	−12.3	0.3	4	0.4
P423A	WT	1.16	0.05	4.6	0.3	−10.9	0.4	4	2.4

Tables show the binding parameters between various *D. rerio* CPAP and STIL constructs obtained from ITC experiments. The measurements of the WT STIL^404–448^—WT CPAP^937–1124^ interaction are identical to each other and identical to those shown in Table 1 and are only presented again to allow easier comparison within each table. Fitting was performed with N as a variable. Constraining N to a fixed value of 1 during fitting produced K_D_ values that were within the experimental error of those tabulated here. In control measurements on wild-type material and a selection of mutants of both CPAP and STIL, the experimental configuration was reversed with CPAP protein titrated into STIL peptide in the ITC cell. These experiments gave similar values for N, K_D_ and ΔH to the standard configuration reported here.

**Table 5.** *D. melanogaster* dSTIL^1–47^-dCPAP^700–901^ crystallisation conditions **DOI:** http://dx.doi.org/10.7554/eLife.01071.019

Crystal	Protein concentration (mg/ml)	Mother liquor	µl protein:µl Mother liquor	µl seed stock
Native	6.18	100 mM MES/imidazole mix pH 6.5, 30 mM MgCl_2_, 30 mM CaCl_2_, 20% ethylene glycol, 10% PEG 8000	0.15:0.05	–
Semet1	5.00	100 mM MES/imidazole mix pH 6.5, 20% ethylene glycol, 10% PEG8000, 0.2 M racemic glutamic acid, 0.2 M glycine, 0.2 M racemic serine, 0.2 M racemic alanine, 0.2 M racemic lysine HCl	0.1:0.1	–
Semet2	5.29	100 mM MES/imidazole mix pH 6.5, 14% ethylene glycol, 7% PEG8000, 30 mM NaNO_3_, 30 mM NaPO_4_, 30 mM NH_4_SO_4_	0.3:0.1	0.05
Semet3	5.29	100 mM MES/imidazole mix pH 6.5, 14% ethylene glycol, 7% PEG8000, 30 mM NaNO_3_, 30 mM NaPO_4_, 30 mM NH_4_SO_4_	0.3:0.1	0.05
Semet4	5.29	100 mM MES/imidazole mix pH 6.5, 16% ethylene glycol, 8% PEG8000, 30 mM NaNO_3_, 30 mM NaPO_4_, 30 mM NH_4_SO_4_	0.3:0.1	0.05

**Table 6.** *D. melanogaster* dSTIL^1–47^-dCPAP^700–901^ SeMet dataset analysis **DOI:** http://dx.doi.org/10.7554/eLife.01071.020

	Semet1-PEAK	SEMET1-LREM	Semet2-Peak	Semet3-PEAK	Semet3-INFL	Semet4-Peak
Beamline	Diamond IO4	Diamond IO4	Diamond IO3	Diamond IO3	Diamond IO3	Diamond IO3
Spacegroup	P1	P1	P1	P1	P1	P1
Wavelength	0.9795	0.9999	0.9792	0.9791	0.9794	0.9791
Unit cell dimensions (Å)	a = 59.31 b = 70.02 c = 70.01 α = 87.65 β = 89.24 γ = 67.37	a = 59.14 b = 70.24 c = 70.13 α = 87.62 β = 89.12 γ = 67.35	a = 58.47 b = 70.15 c = 69.99 α = 87.08 β = 88.41 γ = 67.60	a = 58.56 b = 70.03 c = 70.14 α = 86.93 β = 88.39 γ = 68.09	a = 58.72 b = 70.06 c = 70.28 α = 86.84 β = 88.47 γ = 68.36	a = 59.01 b = 70.17 c = 70.15 α = 87.16 β = 88.64 γ = 67.58
Resolution (Å)	54.74–3.50	64.77–3.50	64.80–3.44	70.04–3.50	70.17–4.60	64.80–3.36
Completeness (overall/inner/outer)	98.1/93.8/98.3	98.4/98.0/98.2	97.8/91.6/93.7	98.3/95.3/97.6	97.8/79.1/89.9	97.6/91.0/97.4
Rmerge (overall/inner/outer)	0.093/0.055/0.118	0.086/0.041/0.238	0.17/0.076/0.518	0.152/0.086/0.336	0.116/0.039/0.189	0.125/0.037/0.433
Rpim (overall/inner/outer)	0.071/0.047/0.136	0.062/0.029/0.172	0.078/0.040/0.229	0.075/0.043/0.184	0.087/0.034/0.141	0.100/0.038/0.323
I/σI (overall/inner/outer)	9.1/16.9/5.6	10.9/24.7/4.9	7.3/18.8/3.5	9.1/27.2/3.6	6.0/20.8/4.6	6.9/22.5/2.7
Multiplicity (overall/inner/outer)	3.9/3.8/3.9	3.9/3.8/3.8	7.0/7.0/7.1	6.0/6.7/5.3	3.5/3.6/3.5	3.5/3.4/3.6
No. unique reflections	12,832	12,884	13,315	12,797	5641	14,461

**Table 7.** Quantification of centriole/centrosome numbers in *dCPAP* or *dSTIL* mutant larval brain cells expressing the indicated WT or mutant constructs **DOI:** http://dx.doi.org/10.7554/eLife.01071.021

Genotype	Number of brains	Total number of cells	Cells with centrosome number (%)			
			0	1	2	3
WT	12	944	2.1	2.6	95.2	0.0
*dCPAP*	8	661	95.2	4.2	0.6	0.0
dCPAP_WT-GFP	9	715	2.8	6.3	90.5	0.4
dCPAP_ΔC-GFP	13	1147	95.1	3.8	1.0	0.0
dCPAP_MC1-GFP	11	968	64.9	30.1	4.8	0.3
dCPAP_MC2-GFP	17	1053	43.5	42.2	14.1	0.3
dCPAP_MC3-GFP	16	1870	4.5	13.3	81.9	0.3
dCPAP_MC1-3-GFP	11	888	90.1	8.0	1.8	0.1
dCPAP_E792V-GFP	9	1015	13.7	31.1	54.8	0.4
*dSTIL*^*169*^	9	846	98.1	1.9	0.0	0.0
*dSTIL*^*719*^	9	980	99.2	0.8	0.0	0.0
dSTIL_WT-GFP	6	424	5.7	9.0	85.1	0.2
dSTIL_ΔN-GFP	13	884	88.1	9.8	1.9	0.1
dSTIL_P11A-GFP	9	1008	11.0	41.0	48.0	0.0
dSTIL_R12A-GFP	9	709	7.0	31.0	62.0	0.0
dSTIL_P11AR12A-GFP	9	727	41.0	43.0	16.0	0.0

### Crystallisation

Native *D. rerio* CPAP^937–1124^ was crystallised in sitting drops in 80 mM Tris pH 8.5, 160 mM MgCl_2_, 20% PEG-4000, 18% Glycerol, 1 mM DTT at 19.5°C. The drops were set up using 1 μl of the protein solution and 0.5 μl of the reservoir solution. Crystals were mounted after 3 days and flash-frozen in liquid nitrogen.

*D. rerio* CPAP^937–1124^ E1021V was crystallised in sitting drops in 80 mM Tris pH 8.5, 160 mM MgCl_2_, 24% PEG-4000, 20% glycerol at 19.5°C. Crystals were mounted after 3 days and flash-frozen in liquid nitrogen.

SeMet *D. rerio* CPAP^937–1124^ crystals were obtained using the sitting drop method with a reservoir solution of 80 mM Tris pH 8.5, 160 mM MgCl_2_, 26% PEG-4000, 18% glycerol, 1 mM DTT at 19.5°C. Drops were set up using 1 μl protein solution and 1 μl of reservoir solution. Native CPAP^937–1124^ crystals were used for streak-seeding into these drops and crystals allowed to grow for 7 days before mounting and flash-freezing them in liquid nitrogen.

Crystals of the complex of *D. rerio* CPAP^937–1124^ and *D. rerio* STIL^408–428^ were initially obtained using the LMB screening set-up with the Clear Strategy 2 pH 8.5 screen (MDL, Newmarket, UK). Crystals were used to streak seed into sitting drops consisting of 1 μl of a reservoir solution of 100 mM Tris pH 8.5, 200 mM CaAcetate, 17% PEG-2000 MME and 1 μl of protein/peptide mixture (0.25 μl protein + 0.75 μl peptide) at 19.5°C. Crystals were grown for 2 days before mounting them in 100 mM Tris pH 8.5, 200 mM CaAcetate, 17% PEG-2000 MME, 25% glycerol and flash-freezing them in liquid nitrogen.

The protein concentrations of *D. rerio* CPAP^937–1124^ used for crystallisations were measured by the Bradford assay with BSA as a standard and were 39.9 mg/ml (apo-CPAP^937–1124^), 46.2 mg/ml (CPAP^937–1124^ E1021V), 30.6 mg/ml (SeMet CPAP^937–1124^) and 81 mg/ml CPAP^937–1124^ (3.7 mM, CPAP/STIL complex). The concentration of STIL^408–428^ (CPAP/STIL complex) was determined by amino acid analysis and was 11.8 mg/ml (3.8 mM).

Native *D. melanogaster* dSTIL^1–47^-dCPAP^700–901^ was crystallised using the sitting drop approach, using the Morpheus screen (Molecular Dimensions). Crystals grew after approximately 3 weeks (Table 5, ‘Native’). Crystals were mounted after approximately 4 weeks. SeMet *D. melanogaster* dSTIL^1–47^-dCPAP^700–901^ was initially crystallised using the Morpheus screen (Molecular Dimensions). Crystals typically grew after 3–4 weeks. Some crystals were used for microseeding of further screens including an optimisation screen. Seed stock was generated using a Seed bead kit (Hampton, Aliso Viejo, CA). Details of crystallisation conditions are shown in Table 5.

### Data collection and processing

Native data were collected as described in Table 2. All *D. rerio* datasets were integrated and scaled using MOSFLM (Leslie and Powell, 2007) and Scala (Evans, 2006) respectively. The *D. rerio* CPAP^937–1124^ structure was solved by MAD in CRANK (Ness et al., 2004; Cowtan, 2006), resulting in clear electron density into which an initial model was built using ArpWarp (Langer et al., 2008). Phenix.refine (Afonine et al., 2005) and REFMAC (Murshudov et al., 2011) were used to refine the model against the native dataset with manual building done in Coot (Emsley and Cowtan, 2004). *D. rerio* CPAP^937–1124^ E1021V was solved by molecular replacement in Phaser (McCoy et al., 2007) using a poly-alanine model derived from the WT model. The model was further built and refined as described for the WT structure. The complex of *D. rerio* CPAP^937–1124^ and *D. rerio* STIL^408–428^ was solved by molecular replacement using Phaser (McCoy et al., 2007) with a distorted model of the *D. rerio* CPAP^937–1124^ WT apo-structure. Refinement yielded clear density for the residues of STIL shown here. The model was further built and refined as described for the other *D. rerio* structures.

*D. melanogaster* dSTIL^1–47^-dCPAP^700–901^ data was scaled using Xia2 (Winter, 2010). Phasing was carried out using all SeMet datasets (Table 6) in autoSHARP (Vonrhein et al., 2007), using SHELXC/D (Sheldrick, 2008) for heavy atom finding, SHARP for site refinement/phasing and SOLOMON (Abrahams and Leslie, 1996) for density modification. This resulted in an experimental density map within which a CHAINSAW (Stein, 2008) model based on the *D. rerio* complex structure could be manually placed, using heavy atom sites as a guide. Experimental density corresponding to the dSTIL peptide could be easily seen. Further refinement cycles allowed the remaining copies of the monomer to be placed and trimmed. Refinement and model building were carried out in autoBUSTER (Bricogne et al., 2011) and Coot (Emsley and Cowtan, 2004) respectively.

### Isothermal calorimetry (ITC) measurements

All ITC measurements were performed using an auto-iTC 200 instrument (GE Healthcare, Little Chalfont, UK) in 50 mM HEPES pH 7.5, 100 mM NaCl at 25°C. Samples were stored by the instrument in 96-well microtiter plates at 5°C prior to loading and performing the titrations. Standard experiments used 19 × 2 μl injections of STIL peptide into CPAP protein preceded by a single 0.5 μl pre-injection. Heat from the pre-injection was not used during fitting. Data were analysed manually in the Origin software package provided by the manufacturer and fit to a single set of binding sites model. All measurements were corrected using control ITC experiments in which the peptide studied was injected into buffer only. The small endothermic heats of injection in these experiments were fitted to a linear function that was subsequently subtracted from the equivalent integrated heats of the peptide–protein binding experiment before fitting. The concentration of CPAP in the cell was typically 40 μM but varied maximally between 20 and 100 μM. The concentration of STIL used in the syringe was typically 700 μM but varied maximally between 600 and 2600 μM depending on the affinity of the peptide interaction being studied.

### In vivo analysis in *Drosophila*

#### Fly stocks and transgenic constructs

The following mutant alleles and stocks were used in this study: *ana2*^*169*^ (here called *dSTIL*^*169*^), *ana2*^*719*^ (here called *dSTIL*^*719*^) (Wang et al., 2011), *sas-4*^*s2214*^ (here called *dCPAP*) (Basto et al., 2006), pUbq-dCPAP_WT-GFP, pUbq-dCPAP_ΔC_1–724_-GFP, pUbq-dCPAP_MC1-GFP, pUbq-dCPAP_MC2-GFP, pUbq-dCPAP_MC3-GFP, pUbq-dCPAP_MC1-3-GFP, pUbq-dCPAP_E792V-GFP, pUbq-dSTIL_WT-GFP, pUbq-dSTIL_ΔN_46–420_-GFP, pUbq-dSTIL_P11A-GFP, pUbq-dSTIL_R12A-GFP, pUbq-dSTIL_P11AR12A-GFP. Transgenic lines contain GFP fused to the C-terminus of dCPAP and dSTIL, respectively, and are expressed from the Ubiquitin promoter, which drives moderate expression in all cell types (Lee et al., 1988). Flies were kept at 25°C, OregonR served as wild-type control.

GFP-tagged full length versions of dCPAP and dSTIL used in this study were made by cloning the full length dCPAP cDNA and the dSTIL cDNA into the pUbq-GFP(C-terminus) destination vector using the Gateway System (Life Technologies, Carlsbad, CA). PCR with dCPAP_WT and dSTIL_WT as template was used to make dCPAP_ΔC_1–724_ and dSTIL_ΔN_46–420_, respectively. Single point mutations and mutation clusters were introduced into full length dCPAP and dSTIL using site-directed mutagenesis (QuickChange II XL/Quick Change Lightening Multi Site-Directed Mutagenesis Kits, Agilent Technologies, Santa Clara, CA). Constructs were injected either by Genetic Services Inc. (Cambridge, MA) or Cambridge DNA Injection Service (Cambridge, UK).

#### Rescue experiments

All constructs were tested for their ability to rescue the uncoordinated phenotype, which is a feature of flies lacking centrioles (Basto et al., 2006). For that purpose, the different versions of dCPAP-GFP and dSTIL-GFP were either crossed into the *dCPAP* or *dSTIL*^*169*^/*dSTIL2*^*719*^ mutant background, and desired pupae were collected from vials and transferred to filter paper (Whatman, Maidstone, UK) for analysis.

#### Immunohistochemistry on third instar larval brains and centrosome quantification

Brains were dissected, squashed, and stained as previously described (Stevens et al., 2009). The following antibodies were used to stain centrosomes in third instar larval brain cells: sheep anti-Centrosomin (Cnn, directed against the N-terminus, 1:1000, [Lucas and Raff, 2007] but raised in sheep), guinea pig anti-Asterless (Asl, 1:500, [Conduit et al., 2010] but raised in guinea pig). Secondary antibodies conjugated to either Alexa Fluor 488 or Alexa Fluor 568 (Life Technologies) were used 1:1000. Hoechst33258 (Life Technologies) was used to visualise DNA (1:5000). Centrosomes were counted on a Zeiss Axioskop 2 microscope (Zeiss, Oberkochen, Germany). Only brain cells in metaphase were scored that did stain for Asl and Cnn. DNA morphology was used to identify cells at the desired stage of the cell cycle. Furthermore, the assessment of centriole loss was performed blind. Microsoft Excel was used to analyse the data. Images were acquired in Metamorph (molecular devices) using a CoolSNAP HQ camera (Photometrics, Tucson, AZ) and processed using ImageJ/Fiji (www.fiji.sc/Fiji, [Schindelin et al., 2012]), Gimp (www.gimp.org/) and Inkscape (www.inkscape.org/) for figure assembly.

#### Western blot analysis

The following primary and secondary antibodies were used: Mouse anti-GFP (1:500, Roche, Basel, Switzerland), mouse anti-actin (1:1000, SIGMA, St. Louis, MO), rabbit anti-dCPAP (1:500) (Basto et al., 2006), anti-mouse HRP (1:3000, GE Healthcare) and anti-rabbit HRP (1:3000, GE Healthcare).

#### Live imaging of embryos

Embryos expressing the different GFP-tagged versions of dCPAP and dSTIL were dechorionated manually and mounted in a Glass Bottom Microwell Dish (MatTek, Ashland, MA) using heptane glue. Embryos were covered with voltalef oil and followed by time-lapse spinning disc microscopy on a Perkin Elmer spinning disc microscope (Perkin Elmer, Waltham, MA). Images were acquired with a charge-coupled Orca ER device camera (Hamamatsu Photonics, Hamamatsu, Japan) using UltraView ERS (Perkin Elmer) and processed and analysed in Velocity (Perkin Elmer).

### *C. elegans* experiments

*C. elegans* strains carrying single-copy *sas-4* transgenes were generated using MosSCI (Frøkjær-Jensen et al., 2008). To render the transgenes RNAi-resistant, a 500 bp region at the 5′ end of the sas-4 genomic sequence was re-encoded. The engineered *sas-4* sequence was cloned into pCFJ151 with the promoter and 3′ UTR from *sas-6*, as well as a C-terminal GFP tag. pCFJ151 contains homology arms that direct transposase-mediated insertion of intervening sequence into the ttTi5606 Mos1 site on Chromosome II. Transgene integration was confirmed by PCR of regions spanning each side of the insertion. The genotypes of the strains used are: unc-119(ed9)III; ltSi85[pOD1550; Psas-6::SAS-4 reencoded::GFP; cb-unc-119(+)]II for WT SAS-4; and unc-119(ed9)III; ltSi177[pOD1551; Psas-6::SAS-4(1-556) reencoded::GFP; cb-unc-119(+)]II for SAS-4^ΔTCP^.

Double-stranded *sas-4* RNA was generated as described (Oegema et al., 2001) using DNA templates prepared by PCR. For experiments to quantify monopolar spindle formation, L4 hermaphrodites were injected with dsRNA and incubated at 20°C for 40 hr prior to dissection for imaging. For lethality assays, worms were maintained at 20°C. L4 hermaphrodites were injected with dsRNA and singled 24 hr post-injection. Adult worms were removed from the plates 48 hr post-injection, and hatched larvae and unhatched embryos were counted 24 hr later.

For light microscopy to identify monopolar or bipolar second division cells, images were acquired using an inverted Zeiss Axio Observer Z1 system with a Yokogawa spinning-disk confocal head (CSU-X1), a 63X 1.4 NA Plan Apochromat objective, and a QuantEM:512SC EMCCD camera (Photometrics). Adult worms were dissected in M9 buffer, and embryos were mounted onto 2% agarose pads for imaging. 11 × 1 μm z-stacks were collected in the GFP channel (100 ms, 20% power, no binning), along with one central DIC section.

#### SAS-4/SAS-5 pull-down experiments

SAS-4 constructs were cloned into a pET21a vector for in vitro transcription/translation. Proteins were expressed using the T7 TNT Quick Coupled Transcription/Translation System (Promega, Fitchburg, WI) with ^35^S-Met labelling.

SAS-5 fragments were cloned into a pRSET-A vector with a C-terminal 6xHis tag. Proteins were expressed in *E. coli* Rosetta2(DE3) cells and purified on Ni-NTA agarose (Qiagen) using standard protocols. For pull-down experiments, proteins were dialysed into 25 mM HEPES, 100 mM NaCl, 20 mM imidazole, 1 mM DTT, 10% sucrose, 0.02% Tween-20, pH 7.4.

SAS-5 fragments were pre-incubated with 20 μl Ni-NTA beads for 45 min at 4°C. 10 μl of the SAS-4 IVTT product was added to the beads with 190 μl buffer and incubated at 4°C for 30 min. The beads were washed with 3 × 200 μl buffer and resuspended in 100 μl SDS-PAGE sample buffer. Samples were run on 10% SDS-PAGE gels and either stained with Coomassie or dried and exposed to a phosphor screen overnight. Phosphor screens were analysed on a Personal Molecular Imager System (Bio-Rad, Hercules, CA).

## Additional files

### Major datasets

The following datasets were generated:

van BreugelM, 2013, Structure of the wild-type TCP10 domain of *Danio rerio* CPAP, http://www.rcsb.org/pdb/search/structidSearch.do?structureId=4bxp, Publicly available at the RCSB Protein Data Bank (http://www.rcsb.org/).

van BreugelM, 2013, Structure of the E1021V mutant of the TCP10 domain of Danio rerio CPAP, http://www.rcsb.org/pdb/search/structidSearch.do?structureId=4bxq, Publicly available at the RCSB Protein Data Bank (http://www.rcsb.org/).

van BreugelM, 2013, Structure of the wild-type TCP10 domain of *Danio rerio* CPAP in complex with a peptide of *Danio rerio* STIL, http://www.rcsb.org/pdb/search/structidSearch.do?structureId=4bxr, Publicly available at the RCSB Protein Data Bank (http://www.rcsb.org/).

CotteeMA, LeaSM, 2013, SAS-4 (dCPAP) TCP domain in complex with a Proline Rich Motif of Ana2 (dSTIL) of *Drosophila melanogaster*, http://www.rcsb.org/pdb/search/structidSearch.do?structureId=4by2, Publicly available at the RCSB Protein Data Bank (http://www.rcsb.org/).

## References

[bib1] Abrahams JP Leslie AG 1996 Methods used in the structure determination of bovine mitochondrial F1 ATPase. Acta Crystallogr D Biol Crystallogr 52:30–42. 10.1107/S0907444995008754 15299723

[bib2] Afonine PV Grosse-Kunstleve RW Adams PD 2005 The Phenix refinement framework. CCP4 Newsletter 42 http://www.ccp4.ac.uk/newsletters/newsletter42/content.html

[bib3] Al-Dosari MS Shaheen R Colak D Alkuraya FS 2010 Novel CENPJ mutation causes Seckel syndrome. J Med Genet 47:411–4. 10.1136/jmg.2009.076646 20522431

[bib4] Arquint C Sonnen KF Stierhof YD Nigg EA 2012 Cell-cycle-regulated expression of STIL controls centriole number in human cells. J Cell Sci 125:1342–52. 10.1242/jcs.099887 22349698

[bib5] Basto R Lau J Vinogradova T Gardiol A Woods CG Khodjakov A 2006 Flies without centrioles. Cell 125:1375–86. 10.1016/j.cell.2006.05.025 16814722

[bib6] Bettencourt-Dias M Hildebrandt F Pellman D Woods G Godinho SA 2011 Centrosomes and cilia in human disease. Trends Genet 27:307–15. 10.1016/j.tig.2011.05.004 21680046 PMC3144269

[bib7] Blachon S Cai X Roberts KA Yang K Polyanovsky A Church A 2009 A proximal centriole-like structure is present in Drosophila spermatids and can serve as a model to study centriole duplication. Genetics 182:133–44. 10.1534/genetics.109.101709 19293139 PMC2674812

[bib8] Bond J Roberts E Springell K Lizarraga SB Scott S Higgins J 2005 A centrosomal mechanism involving CDK5RAP2 and CENPJ controls brain size. Nat Genet 37:353–5. 10.1038/ng1539 15793586

[bib9] Bricogne GBE Brandl M Flensburg C Keller P Paciorek W Roversi PSA 2011 BUSTER version 2.11.4.

[bib10] Brito DA Gouveia SM Bettencourt-Dias M 2012 Deconstructing the centriole: structure and number control. Curr Opin Cell Biol 24:4–13. 10.1016/j.ceb.2012.01.003 22321829

[bib11] Chothia C 1973 Conformation of twisted β-pleated sheets in proteins. J Mol Biol 75:295–302. 10.1016/0022-2836(73)90022-3 4728692

[bib12] Cizmecioglu O Arnold M Bahtz R Settele F Ehret L Haselmann-Weiss U 2010 Cep152 acts as a scaffold for recruitment of Plk4 and CPAP to the centrosome. J Cell Biol 191:731–9. 10.1083/jcb.201007107 21059844 PMC2983070

[bib13] Conduit PT Brunk K Dobbelaere J Dix CI Lucas EP Raff JW 2010 Centrioles regulate centrosome size by controlling the rate of Cnn incorporation into the PCM. Curr Biol 20:2178–86. 10.1016/j.cub.2010.11.011 21145741

[bib14] Cowtan K 2006 The Buccaneer software for automated model building. 1. Tracing protein chains. Acta Crystallogr D Biol Crystallogr 62:1002–11. 10.1107/S0907444906022116 16929101

[bib15] Dammermann A Maddox PS Desai A Oegema K 2008 SAS-4 is recruited to a dynamic structure in newly forming centrioles that is stabilized by the gamma-tubulin-mediated addition of centriolar microtubules. J Cell Biol 180:771–85. 10.1083/jcb.200709102 18299348 PMC2265562

[bib16] Darvish H Esmaeeli-Nieh S Monajemi GB Mohseni M Ghasemi-Firouzabadi S Abedini SS 2010 A clinical and molecular genetic study of 112 Iranian families with primary microcephaly. J Med Genet 47:823–8. 10.1136/jmg.2009.076398 20978018

[bib17] Dobbelaere J Josue F Suijkerbuijk S Baum B Tapon N Raff J 2008 A genome-wide RNAi screen to dissect centriole duplication and centrosome maturation in *Drosophila*. PLOS Biol 6:e224. 10.1371/journal.pbio.0060224 18798690 PMC2535660

[bib18] Dzhindzhev NS Yu QD Weiskopf K Tzolovsky G Cunha-Ferreira I Riparbelli M 2010 Asterless is a scaffold for the onset of centriole assembly. Nature 467:714–8. 10.1038/nature09445 20852615

[bib19] Emsley P Cowtan K 2004 Coot: model-building tools for molecular graphics. Acta Crystallogr D Biol Crystallogr 60:2126–32. 10.1107/S0907444904019158 15572765

[bib20] Evans P 2006 Scaling and assessment of data quality. Acta Crystallogr D Biol Crystallogr 62:72–82. 10.1107/S0907444905036693 16369096

[bib21] Frøkjær-Jensen C Davis MW Hopkins CE Newman BJ Thummel JM Olesen SP 2008 Single-copy insertion of transgenes in *Caenorhabditis elegans*. Nat Genet 40:1375–83. 10.1038/ng.248 18953339 PMC2749959

[bib22] Glaser F Pupko T Paz I Bell RE Bechor-Shental D Martz E 2003 ConSurf: identification of functional regions in proteins by surface-mapping of phylogenetic information. Bioinformatics 19:163–4. 10.1093/bioinformatics/19.1.163 12499312

[bib23] Gonczy P 2012 Towards a molecular architecture of centriole assembly. Nat Rev Mol Cell Biol 13:425–35. 10.1038/nrm3373 22691849

[bib24] Gul A Hassan MJ Hussain S Raza SI Chishti MS Ahmad W 2006 A novel deletion mutation in *CENPJ* gene in a Pakistani family with autosomal recessive primary microcephaly. J Hum Genet 51:760–4. 10.1007/s10038-006-0017-1 16900296

[bib25] Han YG Alvarez-Buylla A 2010 Role of primary cilia in brain development and cancer. Curr Opin Neurobiol 20:58–67. 10.1016/j.conb.2009.12.002 20080044 PMC2829308

[bib26] Hatch EM Kulukian A Holland AJ Cleveland DW Stearns T 2010 Cep152 interacts with Plk4 and is required for centriole duplication. J Cell Biol 191:721–9. 10.1083/jcb.201006049 21059850 PMC2983069

[bib27] Hiraki M Nakazawa Y Kamiya R Hirono M 2007 Bld10p constitutes the cartwheel-spoke tip and stabilizes the 9-fold symmetry of the centriole. Curr Biol 17:1778–83. 10.1016/j.cub.2007.09.021 17900905

[bib28] Hsu WB Hung LY Tang CJ Su CL Chang Y Tang TK 2008 Functional characterization of the microtubule-binding and -destabilizing domains of CPAP and d-SAS-4. Exp Cell Res 314:2591–602. 10.1016/j.yexcr.2008.05.012 18586240

[bib29] Hung LY Tang CJ Tang TK 2000 Protein 4.1 R-135 interacts with a novel centrosomal protein (CPAP) which is associated with the gamma-tubulin complex. Mol Cell Biol 20:7813–25. 10.1128/MCB.20.20.7813-7825.2000 11003675 PMC86375

[bib30] Hussain MS Baig SM Neumann S Nurnberg G Farooq M Ahmad I 2012 A truncating mutation of CEP135 causes primary microcephaly and disturbed centrosomal function. Am J Hum Genet 90:871–8. 10.1016/j.ajhg.2012.03.016 22521416 PMC3376485

[bib31] Islam SD Pilder SH Decker CL Cebra-Thomas JA Silver LM 1993 The human homolog of a candidate mouse t complex responder gene: conserved motifs and evolution with punctuated equilibria. Hum Mol Genet 2:2075–9. 10.1093/hmg/2.12.2075 8111376

[bib32] Kalay E Yigit G Aslan Y Brown KE Pohl E Bicknell LS 2011 CEP152 is a genome maintenance protein disrupted in Seckel syndrome. Nat Genet 43:23–6. 10.1038/ng.725 21131973 PMC3430850

[bib33] Kay BK Williamson MP Sudol M 2000 The importance of being proline: the interaction of proline-rich motifs in signaling proteins with their cognate domains. FASEB J 14:231–41 10657980

[bib34] Kernan M Cowan D Zuker C 1994 Genetic dissection of mechanosensory transduction: mechanoreception-defective mutations of *Drosophila*. Neuron 12:1195–206. 10.1016/0896-6273(94)90437-5 8011334

[bib35] Kim MK Dudognon C Smith S 2012 Tankyrase 1 regulates centrosome function by controlling CPAP stability. EMBO Rep 13:724–32. 10.1038/embor.2012.86 22699936 PMC3410387

[bib36] Kirkham M Muller-Reichert T Oegema K Grill S Hyman AA 2003 SAS-4 is a *C. elegans* centriolar protein that controls centrosome size. Cell 112:575–87. 10.1016/S0092-8674(03)00117-X 12600319

[bib37] Kitagawa D Kohlmaier G Keller D Strnad P Balestra FR Fluckiger I 2011a Spindle positioning in human cells relies on proper centriole formation and on the microcephaly proteins CPAP and STIL. J Cell Sci 124:3884–93. 10.1242/jcs.089888 22100914

[bib38] Kitagawa D Vakonakis I Olieric N Hilbert M Keller D Olieric V 2011b Structural basis of the 9-fold symmetry of centrioles. Cell 144:364–75. 10.1016/j.cell.2011.01.008 21277013 PMC3089914

[bib39] Kleylein-Sohn J Westendorf J Le Clech M Habedanck R Stierhof YD Nigg EA 2007 Plk4-induced centriole biogenesis in human cells. Dev Cell 13:190–202. 10.1016/j.devcel.2007.07.002 17681131

[bib40] Kohlmaier G Loncarek J Meng X McEwen BF Mogensen MM Spektor A 2009 Overly long centrioles and defective cell division upon excess of the SAS-4-related protein CPAP. Curr Biol 19:1012–8. 10.1016/j.cub.2009.05.018 19481460 PMC2993638

[bib41] Konarev PV Volkov VV Sokolova AV Koch MHJ Svergun DI 2003 PRIMUS: a windows PC-based system for small-angle scattering data analysis. J Appl Cryst 36:1277–82. 10.1107/S0021889803012779

[bib42] Langer G Cohen SX Lamzin VS Perrakis A 2008 Automated macromolecular model building for X-ray crystallography using ARP/wARP version 7. Nat Protoc 3:1171–9. 10.1038/nprot.2008.91 18600222 PMC2582149

[bib43] Leal GF Roberts E Silva EO Costa SM Hampshire DJ Woods CG 2003 A novel locus for autosomal recessive primary microcephaly (MCPH6) maps to 13q12.2. J Med Genet 40:540–2. 10.1136/jmg.40.7.540 12843329 PMC1735531

[bib44] Lee HS Simon JA Lis JT 1988 Structure and expression of ubiquitin genes of *Drosophila melanogaster*. Mol Cell Biol 8:4727–35 2463465 10.1128/mcb.8.11.4727-4735.1988 PMC365564

[bib45] Leidel S Gonczy P 2003 SAS-4 is essential for centrosome duplication in *C. elegans* and is recruited to daughter centrioles once per cell cycle. Dev Cell 4:431–9. 10.1016/S1534-5807(03)00062-5 12636923

[bib46] Leslie AGW Powell HR 2007 Processing diffraction data with Mosflm. Evolving methods for macromolecular crystallography. 245:41–51

[bib47] Leung GC Hudson JW Kozarova A Davidson A Dennis JW Sicheri F 2002 The Sak polo-box comprises a structural domain sufficient for mitotic subcellular localization. Nat Struct Biol 9:719–24. 10.1038/nsb848 12352953

[bib48] Lin YC Chang CW Hsu WB Tang CJ Lin YN Chou EJ 2013 Human microcephaly protein CEP135 binds to hSAS-6 and CPAP, and is required for centriole assembly. EMBO J 32:1141–54. 10.1038/emboj.2013.56 23511974 PMC3630357

[bib49] Lucas EP Raff JW 2007 Maintaining the proper connection between the centrioles and the pericentriolar matrix requires Drosophila centrosomin. J Cell Biol 178:725–32. 10.1083/jcb.200704081 17709428 PMC2064537

[bib50] Lukinavičius G Lavogina D Orpinell M Umezawa K Reymond L Garin N 2013 Selective chemical crosslinking reveals a Cep57-Cep63-Cep152 centrosomal complex. Curr Biol 23:265–70. 10.1016/j.cub.2012.12.030 23333316

[bib51] Makabe K McElheny D Tereshko V Hilyard A Gawlak G Yan S 2006 Atomic structures of peptide self-assembly mimics. Proc Natl Acad Sci USA 103:17753–8. 10.1073/pnas.0606690103 17093048 PMC1693819

[bib52] McCoy AJ Grosse-Kunstleve RW Adams PD Winn MD Storoni LC Read RJ 2007 *Phaser* crystallographic software. J Appl Crystallogr 40:658–74. 10.1107/S0021889807021206 19461840 PMC2483472

[bib53] McIntyre RE Lakshminarasimhan Chavali P Ismail O Carragher DM Sanchez-Andrade G Forment JV 2012 Disruption of mouse Cenpj, a regulator of centriole biogenesis, phenocopies Seckel syndrome. PLOS Genet 8:e1003022. 10.1371/journal.pgen.1003022 23166506 PMC3499256

[bib54] Megraw TL Sharkey JT Nowakowski RS 2011 Cdk5rap2 exposes the centrosomal root of microcephaly syndromes. Trends Cell Biol 21:470–80. 10.1016/j.tcb.2011.04.007 21632253 PMC3371655

[bib55] Mennella V Keszthelyi B McDonald KL Chhun B Kan F Rogers GC 2012 Subdiffraction-resolution fluorescence microscopy reveals a domain of the centrosome critical for pericentriolar material organization. Nat Cell Biol 14:1159–68. 10.1038/ncb2597 23086239 PMC3767400

[bib56] Murshudov GN Skubak P Lebedev AA Pannu NS Steiner RA Nicholls RA 2011 REFMAC5 for the refinement of macromolecular crystal structures. Acta Crystallogr D Biol Crystallogr 67:355–67. 10.1107/S0907444911001314 21460454 PMC3069751

[bib57] Murzin AG 1992 Structural principles for the propeller assembly of beta-sheets: the preference for seven-fold symmetry. Proteins 14:191–201. 10.1002/prot.340140206 1409568

[bib58] Nakazawa Y Hiraki M Kamiya R Hirono M 2007 SAS-6 is a cartwheel protein that establishes the 9-fold symmetry of the centriole. Curr Biol 17:2169–74. 10.1016/j.cub.2007.11.046 18082404

[bib59] Ness SR de Graaff RA Abrahams JP Pannu NS 2004 CRANK: new methods for automated macromolecular crystal structure solution. Structure 12:1753–61. 10.1016/j.str.2004.07.018 15458625

[bib60] Nigg EA Raff JW 2009 Centrioles, centrosomes, and cilia in health and disease. Cell 139:663–78. 10.1016/j.cell.2009.10.036 19914163

[bib61] O’Connell KF Caron C Kopish KR Hurd DD Kemphues KJ Li Y 2001 The *C. elegans zyg-1* gene encodes a regulator of centrosome duplication with distinct maternal and paternal roles in the embryo. Cell 105:547–58. 10.1016/S0092-8674(01)00338-5 11371350

[bib62] Oegema K Desai A Rybina S Kirkham M Hyman AA 2001 Functional analysis of kinetochore assembly in *Caenorhabditis elegans*. J Cell Biol 153:1209–26. 10.1083/jcb.153.6.1209 11402065 PMC2192036

[bib63] Pelletier L O’Toole E Schwager A Hyman AA Müller-Reichert T 2006 Centriole assembly in *Caenorhabditis elegans*. Nature 444:619–23. 10.1038/nature05318 17136092

[bib64] Qiao R Cabral G Lettman MM Dammermann A Dong G 2012 SAS-6 coiled-coil structure and interaction with SAS-5 suggest a regulatory mechanism in *C. elegans* centriole assembly. EMBO J 31:4334–47. 10.1038/emboj.2012.280 23064147 PMC3501224

[bib65] Roque H Wainman A Richens J Kozyrska K Franz A Raff JW 2012 *Drosophila* Cep135/Bld10 maintains proper centriole structure but is dispensable for cartwheel formation. J Cell Sci 125:5881–6. 10.1242/jcs.113506 22976301

[bib66] Schindelin J Arganda-Carreras I Frise E Kaynig V Longair M Pietzsch T 2012 Fiji: an open-source platform for biological-image analysis. Nat Methods 9:676–82. 10.1038/nmeth.2019 22743772 PMC3855844

[bib67] Schmidt TI Kleylein-Sohn J Westendorf J Le Clech M Lavoie SB Stierhof YD 2009 Control of centriole length by CPAP and CP110. Curr Biol 19:1005–11. 10.1016/j.cub.2009.05.016 19481458

[bib68] Sheldrick GM 2008 A short history of SHELX. Acta Crystallogr A 64:112–22. 10.1107/S0108767307043930 18156677

[bib69] Siller KH Doe CQ 2009 Spindle orientation during asymmetric cell division. Nat Cell Biol 11:365–74. 10.1038/ncb0409-365 19337318

[bib70] Slevin LK Nye J Pinkerton DC Buster DW Rogers GC Slep KC 2012 The structure of the plk4 cryptic polo box reveals two tandem polo boxes required for centriole duplication. Structure 20:1905–17. 10.1016/j.str.2012.08.025 23000383 PMC3496063

[bib71] Sonnen KF Schermelleh L Leonhardt H Nigg EA 2012 3D-structured illumination microscopy provides novel insight into architecture of human centrosomes. Biol Open 1:965–76. 10.1242/bio.20122337 23213374 PMC3507176

[bib72] Stein N 2008 *CHAINSAW*: a program for mutating pdb files used as templates in molecular replacement. J Appl Cryst 41:641–3. 10.1107/S0021889808006985

[bib73] Stevens NR Dobbelaere J Brunk K Franz A Raff JW 2010a *Drosophila* Ana2 is a conserved centriole duplication factor. J Cell Biol 188:313–23. 10.1083/jcb.200910016 20123993 PMC2819680

[bib74] Stevens NR Dobbelaere J Wainman A Gergely F Raff JW 2009 Ana3 is a conserved protein required for the structural integrity of centrioles and basal bodies. J Cell Biol 187:355–63. 10.1083/jcb.200905031 19948479 PMC2779252

[bib75] Stevens NR Roque H Raff JW 2010b DSas-6 and Ana2 coassemble into tubules to promote centriole duplication and engagement. Dev Cell 19:913–9. 10.1016/j.devcel.2010.11.010 21145506 PMC4159445

[bib76] Svergun DI Barberato C Koch MHJ 1995 *CRYSOL*– a program to evaluate x-ray solution scattering of biological macromolecules from atomic coordinates. J Appl Cryst 28:768–73. 10.1107/S0021889895007047

[bib77] Tang CJ Fu RH Wu KS Hsu WB Tang TK 2009 CPAP is a cell-cycle regulated protein that controls centriole length. Nat Cell Biol 11:825–31. 10.1038/ncb1889 19503075

[bib78] Tang CJ Lin SY Hsu WB Lin YN Wu CT Lin YC 2011 The human microcephaly protein STIL interacts with CPAP and is required for procentriole formation. EMBO J 30:4790–804. 10.1038/emboj.2011.378 22020124 PMC3243611

[bib79] Thornton GK Woods CG 2009 Primary microcephaly: do all roads lead to Rome? Trends Genet 25:501–10. 10.1016/j.tig.2009.09.011 19850369 PMC2816178

[bib80] van Breugel M Hirono M Andreeva A Yanagisawa HA Yamaguchi S Nakazawa Y 2011 Structures of SAS-6 suggest its organization in centrioles. Science 331:1196–9. 10.1126/science.1199325 21273447

[bib81] Vonrhein C Blanc E Roversi P Bricogne G 2007 Automated structure solution with autoSHARP. Methods Mol Biol 364:215–30. 10.1385/1-59745-266-1:215 17172768

[bib82] Vulprecht J David A Tibelius A Castiel A Konotop G Liu F 2012 STIL is required for centriole duplication in human cells. J Cell Sci 125:1353–62. 10.1242/jcs.104109 22349705

[bib83] Wang C Li S Januschke J Rossi F Izumi Y Garcia-Alvarez G 2011 An ana2/ctp/mud complex regulates spindle orientation in *Drosophila* neuroblasts. Dev Cell 21:520–33. 10.1016/j.devcel.2011.08.002 21920316

[bib84] Winter G 2010 xia2: an expert system for macromolecular crystallography data reduction. J Appl Cryst 43:186–90. 10.1107/S0021889809045701

